# Transcriptomic studies reveal a key metabolic pathway contributing to a well-maintained photosynthetic system under drought stress in foxtail millet (*Setaria italica* L.)

**DOI:** 10.7717/peerj.4752

**Published:** 2018-05-08

**Authors:** Weiping Shi, Jingye Cheng, Xiaojie Wen, Jixiang Wang, Guanyan Shi, Jiayan Yao, Liyuan Hou, Qian Sun, Peng Xiang, Xiangyang Yuan, Shuqi Dong, Pingyi Guo, Jie Guo

**Affiliations:** 1 College of Agronomy, Shanxi Agricultural University, Taigu, China; 2 Biotechnology Research Institute, Chinese Academy of Agricultural Sciences, Beijing, China; 3 College of Agronomy, Yangzhou University, Yangzhou, China; 4 Industrial Crop Institute, Shanxi Academy of Agricultural Sciences, Fenyang, China; 5 Department of Next Generation Sequencing, Vazyme Biotech Company Ltd., Nanjing, China

**Keywords:** Co-regulation network, Drought tolerance, Photosynthesis, RNA sequencing, Foxtail millet (*Setaria italica* L.)

## Abstract

Drought stress is one of the most important abiotic factors limiting crop productivity. A better understanding of the effects of drought on millet (*Setaria italica* L.) production, a model crop for studying drought tolerance, and the underlying molecular mechanisms responsible for drought stress responses is vital to improvement of agricultural production. In this study, we exposed the drought resistant F_1_ hybrid, M79, and its parental lines E1 and H1 to drought stress. Subsequent physiological analysis demonstrated that M79 showed higher photosynthetic energy conversion efficiency and drought tolerance than its parents. A transcriptomic study using leaves collected six days after drought treatment, when the soil water content was about ∼20%, identified 3066, 1895, and 2148 differentially expressed genes (DEGs) in M79, E1 and H1 compared to the respective untreated controls, respectively. Further analysis revealed 17 Gene Ontology (GO) enrichments and 14 Kyoto Encyclopedia of Genes and Genomes (KEGG) pathways in M79, including photosystem II (PSII) oxygen-evolving complex, peroxidase (POD) activity, plant hormone signal transduction, and chlorophyll biosynthesis. Co-regulation analysis suggested that these DEGs in M79 contributed to the formation of a regulatory network involving multiple biological processes and pathways including photosynthesis, signal transduction, transcriptional regulation, redox regulation, hormonal signaling, and osmotic regulation. RNA-seq analysis also showed that some photosynthesis-related DEGs were highly expressed in M79 compared to its parental lines under drought stress. These results indicate that various molecular pathways, including photosynthesis, respond to drought stress in M79, and provide abundant molecular information for further analysis of the underlying mechanism responding to this stress.

## Introduction

Drought is one of the main abiotic stresses that affect global crop production. It also severely influences metabolism and growth of many crops (Reynolds & Tuberosa, 2008; Zhao & Running, 2011). Foxtail millets (*Setaria italica* L.) is a widely cultivated, dryland crop with superior drought tolerance and higher water use efficiency (WUE) compared to other crops such as corn, sorghum, and wheat (Lata, Gupta & Prasad, 2013). Foxtail millet has a small genome, fast growth, and rich germplasm resources, making it a model crop for stress tolerance research (Li & Brutnell, 2011; Muthamilarasan & Prasad, 2015).

Due to the lack of complete reference genome sequence, previous studies used suppression subtractive hybridization (SSH) and complementary DNA-amplified fragment length polymorphism (cDNA-AFLP) to explore drought-stress response genes in millets (Zhang et al., 2007; Puranik et al., 2011). However, genomic research in millet became easier after whole genome sequencing and annotation of the Zhanggu and Yugu1 varieties are available (Bennetzen et al., 2012; Zhang et al., 2012). RNA-seq technology has been widely used to study how stress factors affect transcriptome in crops such as maize (Zhang et al., 2013), wheat (Camilios-Neto et al., 2014), rice (Zhou et al., 2016), sorghum (Fracasso, Trindade & Amaducci, 2016), and foxtail millet (Qi et al., 2013; Yadav, Khan & Prasad, 2015; Yi, Chen & Yu, 2015; Wang et al., 2016b). Using transcriptomic analysis, Qi et al. (2013) identified 2824 genes and 215 miRNAs that respond to osmotic stress; while Yadav, Khan & Prasad (2015) found 55 known and 136 new miRNAs differentially expressed genes in two millet varieties after treating plants with 20% PEG-6000 to induce dehydration stress. Using the parallel analysis of RNA ends (PARE) and RNA-seq, Yi, Chen & Yu (2015) identified four decay modes of millet mRNA in response to drought stress. Wang et al. (2016b) found that the millet variety An04-4783 expressed 81 known miRNAs and 72 new miRNAs under drought stress. These reports provide important information on drought responsive mechanisms and related regulatory networks in millet.

Millet is a C4 crop, and photosynthesis is the most important to its carbon metabolisms (Lata, Gupta & Prasad, 2013). However, drought stress changes the intracellular environment, which results in decreased electron transfer rates, uncoupling of photosynthetic phosphorylation, increased hydrolysis, and reduced chlorophyll biosynthesis and photosynthetic enzyme activity. The chloroplast is the central organelle that produces reactive oxygen species (ROS), whereas accumulation of ROS may cause oxidative damage and inhibit photosynthesis (Gollan, Tikkanen & Aro, 2015; Exposito-Rodriguez et al., 2017). However, crops are capable of coping with stress through various mechanisms, including osmotic adjustment, accumulation of protective proteins, and antioxidant defense systems (Cui et al., 2016; Gürel et al., 2016). These regulatory pathways crosstalk with each other to form a drought-defensive network that allows plants to maintain photosynthesis under drought stress, and ensures biomass accumulation and eventually high yield (Redillas et al., 2012; Hu & Xiong, 2014; He et al., 2016). Therefore, the ability of crops to maintain photosynthesis under drought stress is an important indicator of drought tolerance (Ma et al., 2016). However, how the expression of photosynthetic genes in response to drought stress is related to drought tolerance remains poorly studied (Ambavaram et al., 2014).

In this study, we employed RNA-seq to investigate the transcriptomic changes between the hybrid M79 and its parental lines in response to drought stress with the aims at identifying DEGs related to drought tolerance, and understanding the associated molecular mechanisms and metabolic pathways in millet. The results may facilitate establishment of a molecular photosynthesis regulatory network of millet under drought stress to lay a foundation for molecular breeding of drought tolerant millet varieties.

## Materials and Methods

### Materials and experimental design

The materials used in this study were E1 (maternal line), H1 (paternal line) and their F_1_ hybrid M79, a drought resistant variety. H1 is a drought-tolerant cultivar released from the Shanxi Academy of Agricultural Sciences, and has been sporadically grown in arid and barren areas during the past decade. E1 developed from the same institute has been an important parental line for many varieties currently grown in China.

Millet seeds were surface sterilized with 0.5% NaClO, washed three times with ddH_2_O and sown in pots (diameter: 8 cm, height: 10 cm, 15 seeds per pot) filled with peat and nutrient soil (1:1). After the pots were kept in a growth chamber (light/darkness: 16h/8 h, temperature: 30 °C/22 °C) for seven days, five healthy plants were maintained in each pot by removing extra plants. After three weeks, the plants at six-leaf stage were treated with drought stress. Each genotype with 100 plants were divided into the control group (M79_CK, E1_CK, and H1_CK) with regular watering, and the drought group (M79_DR, E1_DR, and H1_DR) without watering. For the latter group, the soil gravimetric water content was monitored using a soil moisture analyzer TDR300 (Spectrum, Aurora, IL, USA). At the 5th, 6th, and 7th days after stopping watering, the soil gravimetric water content dropped to 26.1%, 20.3%, and 14.6% of field capacity, respectively.

**Figure 1:**
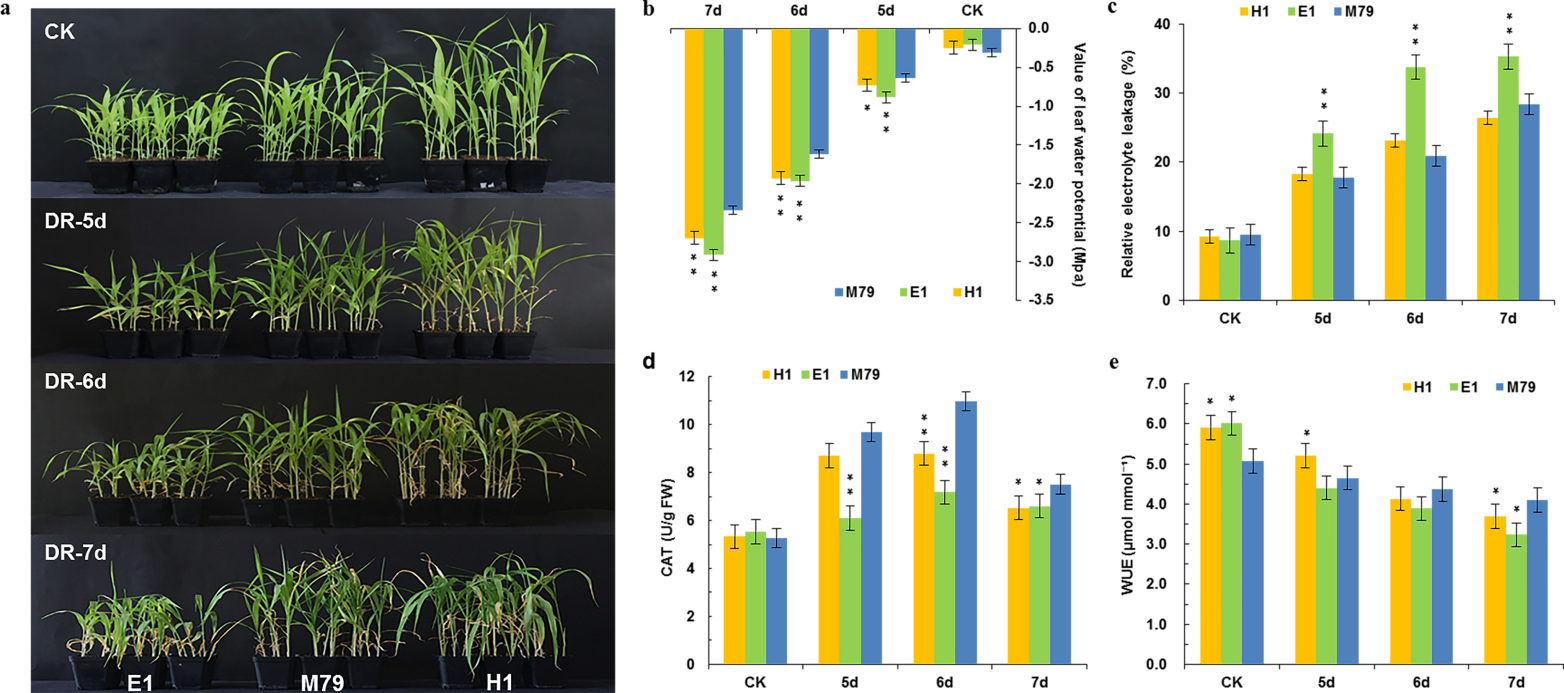
Morphological and physiological analysis of M79, E1 and H1 before and after drought treatment. (A) Phenotypes of M79, E1 and H1 seedlings under normal conditions and 5–7 days after drought stress (photo credit: Weiping Shi); (B) LWP of M79, E1 and H1 under normal conditions and 5–7 days after drought stress; (C) REL in M79, E1 and H1 at 5–7 days after drought stress; (D) CAT activities of M79, E1 and H1 under normal condition and 5–7 days after drought stress; (E) WUE of M79, E1 and H1 under normal condition and 5–7 days after drought stress. Each column represents the mean ± SD (five replicates); *, Significance levels in comparison to M79 were determined by t-tests (* *P* < 0.05, ** *P* < 0.01).

Samples were collected on the 6th day when the soil water content was about 20% (Tang et al., 2017). At this stage, the water potential in M79 leaves was significantly higher than those of the parental lines (Fig. 1B). Plants from the control group (soil water content about 55%) were also sampled at the same stage. Samples were immediately wrapped in foil and frozen in liquid nitrogen, and then kept in a −80 °C freezer. The top second leaf was used as the test materials for all treatments with the upper half for transcriptome sequencing and the lower half for measuring physiological indicators including catalase (CAT) and relative electrolyte leakage (REL). All samples were biologically duplicated three times. The top second leaves from different plants of the same treatments were used for measuring leaf water potential (LWP), photosynthetic rate (A), transpiration rate (E), maximum energy conversion efficiency in PSII centers (Fv/Fm), and quantum yield of PSII electron transport (ΦPSII). The back of each tested leaf was labeled before measuring LWP, A, E, Fv/Fm and ΦPSII to facilitate the next measurement. Each measurement was repeated five times using fully expanded, uninjured leaves.

### Measurement of drought-related physiological changes

Plasma membrane damage of millet leaves was determined as previously reported (Cao et al., 2007), and REL was used to measure the extent of damage to the plasma membrane. CAT quantification was performed following Bonnecarrère et al. (2011). LWP was measured using a Psypro plant water potential meter (WESCOR, Logan, UT, USA). A, E, Fv/Fm, ΦPSII were measured using a LI-6800 portable photosynthesis system (LI-COR, Lincoln, NE, USA) as described by Lowry et al. (2015). Light intensity, CO_2_ concentration, and air flow rate were set to 800 µmol m^−2^ s^−1^, 400 µmol mol^−1^, and 500 µmol s^−1^, respectively. Measurements were carried out from 8:30 - 11:30 am on each day. WUE (µmol mmol^−1^) was calculated by: WUE = A (µmol m^−2^ s^−1^)/E (mol m^−2^ s^−1^)/1000.

### RNA extraction, cDNA library construction, and transcriptome sequencing

RNA samples were prepared from 18 harvests (2 treatments × 3 genotypes × 3 biological replicates) using Trizol^®^ reagent (Invitrogen, Waltham, MA, USA) for subsequent RNA-seq analysis. Quality and concentration of RNA were determined by agarose gel electrophoresis and a NanoDrop 2000 spectrophotometer (Thermo Fisher Scientific, Waltham, MA, USA). A more accurate RNA quantification was performed by using an Agilent 2100 Bioanalyzer RNA Nano chip (Agilent Technologies, Waldbronn, Germany).

Messenger RNA from each sample was enriched using mRNA Capture Beads and then fragmented at high temperature in the presence of metal ions. Using mRNA as a template, the first strand cDNA was synthesized using random hexamers, followed by synthesis of the second strand cDNA, and then purification of the double-stranded cDNA using VAHTS™ DNA Clean Beads. After end repair and A-tailing, purified double-strand cDNA was ligated to the sequencing adapter and sorted using VAHTS™ DNA Clean Beads to get 300–400 bp fragments. Finally, PCR amplification was performed, and PCR products were purified with VAHTS™ DNA Clean Beads to generate the final libraries. Library concentrations were assayed using Qubit 3.0, and the library inserts were subsequently examined using an Agilent Bioanalyzer 2100 system (Agilent Technologies, Santa Clara, CA, USA) and further quantified using the ABI Step One Plus Real-Time PCR system. Finally, the libraries were pooled and sequenced on a HiSeq X Ten (Illumina, San Diego, CA, USA) platform using PE150 mode by Nanjing Vazyme Biotech Company, Ltd.

### RNA-seq data analysis

Clean reads extracted from raw reads using Tophat2 (v2.0.13) were compared with the reference genome *Setaria italica* V2.2 (phytozome.jgi.doe.gov) to get mapped reads (Kim et al., 2013). Based on the available data, we also performed analyses on gene saturation, homogeneity of sequencing, the proportions of mapped reads in genomic exons, introns, and intergenic regions, and correlation analysis between replicates.

Gene expression analysis was performed using Cufflinks v2.2.1. Transcriptome reads aligned to the reference genome were quantified and normalized to fragments per kilobase of transcript per million fragments mapped (FPKM), differences between drought-treated, and the control FPKM values were compared using the software Cuffdiff v2.2.1 (Trapnell et al., 2010). The thresholds of DEGs were set as FDR ≤ 0.05 and |log_2_ FoldChange|≥ 1.

### Functional annotation, pathway analysis, clustered heat map, and co-regulation network analysis

All DEGs were mapped to terms in the GO database (http://www.geneontology.org/), and the number of genes per term was calculated. Based on the GO:: TermFinder, the GO enrichment of the DEGs was performed using a hypergeometric test with a corrected *FDR* < 0.05 as a threshold (Boyle et al., 2004). A biological pathway analysis of DEGs was performed using KEGG (http://www.genome.jp/kegg/), and significance was calculated by hypergeometric distribution with a corrected *FDR* < 0.05 as the threshold (Kanehisa et al., 2008).

To generate a clustered heat map, expression data were converted using the formula log_2_ (FPKM + 1), and the map was drawn using the heatmap 2 function in the R/Bioconductor package gplots (Warnes, 2016).

Co-regulation network analysis was conducted by using Cytoscape (v3.4.0) to plot the co-regulation network with the Pearson correlation coefficient setting |PCC| ≥ 0.93 (Saito et al., 2012).

### qRT-PCR

To verify RNA-seq data, DEGs were confirmed by qRT-PCR following Livak & Schmittgen (2001). Primers were designed (Table S2) based on gene sequences from *Setaria italica* V2.2 (phytozome.jgi.doe.gov) using Primer 3 (http://frodo.wi.mit.edu/). Quantitative PCR was performed using a SYBR^®^ Green PCR Master Mix Kit (Applied Biosystems, Foster City, CA, USA) and an ABI7900 system. The 2^−ΔΔ*CT*^ method was used to calculate relative gene expression. Correlation between RNA-seq and qRT-PCR was analyzed using SPSS 22.0 software (IBM, USA).

## Results

### Phenotypes of M79 and its parental lines under drought conditions

After drought treatment for five days, seedlings of M79 and its parental lines showed no obvious differences. However, after 6 or 7 days of drought stress, leaves in most E1 and H1 plants exhibited curling and withering, whereas M79 appeared normal (Fig. 1A). Physiological analysis of those plants before and after drought treatment showed that LWP decreased sharply after drought stress. On the 5th day of drought treatment, the water potentials of E1 and H1 significantly declined (*P* < 0.01 and 0.05, respectively) in comparison to M79 (Fig. 1B). We used REL to analyze the amounts of damage after drought. From the 5th day, REL of E1 was significantly higher than that of M79 (*P* < 0.01), while the REL of M79 on the 6th day was the lowest among the three genotypes (20.87%) (Fig. 1C). The CAT activity of M79 was the highest (10.97) on the 6th day (Fig. 1D), which was significantly higher than that of E1 and H1. Although the WUE of all genotypes decreased after drought stress, M79 exhibited a significantly higher WUE on the 6th and 7th days (4.37 and 4.10, respectively) compared to E1 and H1 (Fig. 1E). These results demonstrated that M79 had better tolerance to drought stress than its parental lines as shown by phenotypical and physiological indexes.

### RNA-seq data export, quality control, and sequence alignment

Leaves of M79 and its parental lines were sampled for transcriptomic sequencing six days after drought stress. A total of 18 libraries were constructed and sequenced using the HiSeq X Ten sequencing platform, and generated 8.55 × 10^8^ raw reads. After removing the linker and low-quality data, we obtained 8.17 × 10^8^ (95.57%) clean reads, consisting of about 122.58 Gb of clean data, and representing an average of 4.54 × 10^7^ clean reads, i.e., about 6.81 Gb of valid data per sample. Phred mass fraction Q30 (error rate 0.1%) ranged from 86.38 to 88.22%, with an average GC content of 56.30%. We aligned 93.13 to 94.32% of the valid data to the reference genome (Table S1). The FPKM density distribution (Fig. S1A) and FPKM box diagram (Fig. S1B) suggested that the density of the detected genes followed a standard normal distribution. These results indicated high quality and reasonable reproducibility of our sequence data.

### Validation by qRT-PCR

**Figure 2:**
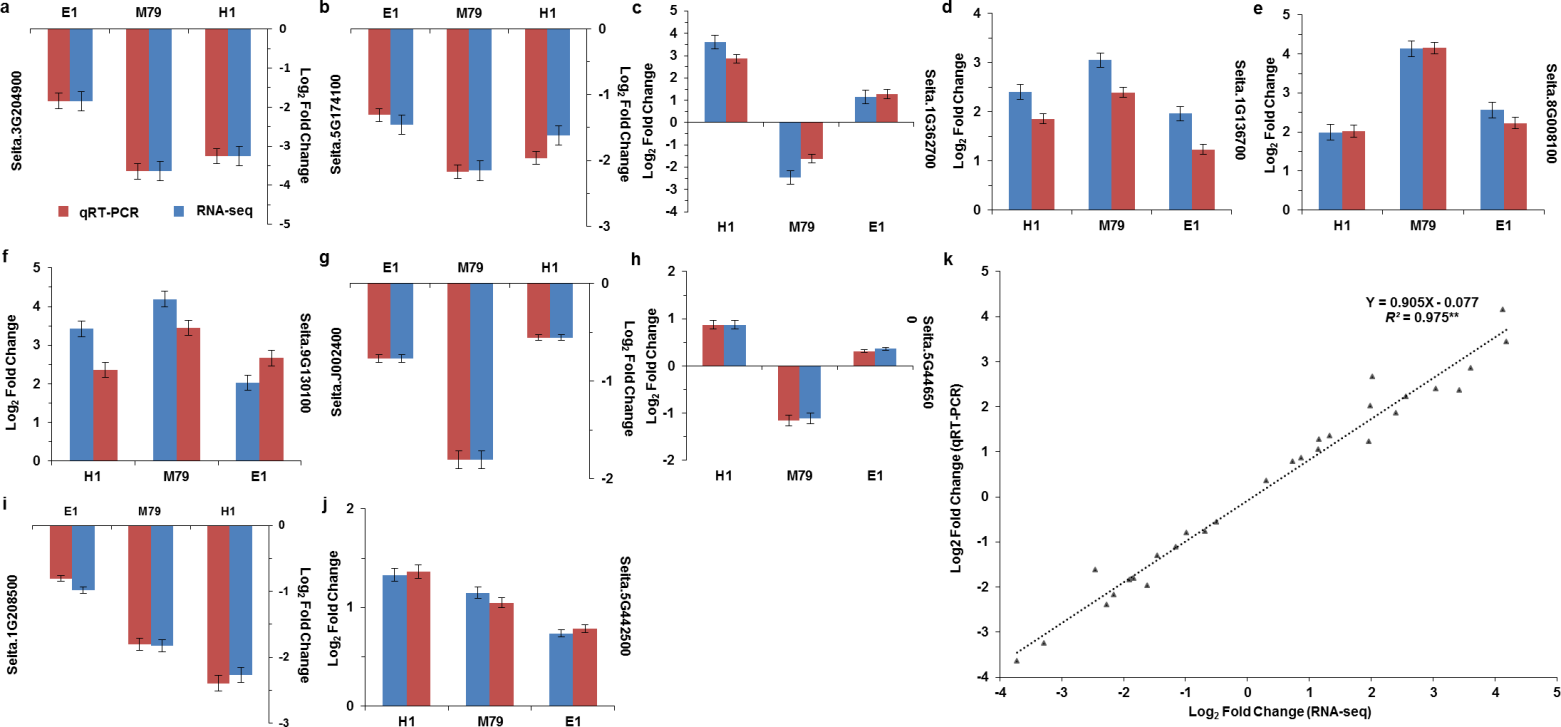
Correlation analysis of RNA-seq and qRT-PCR results. (A–J) Expression levels of 10 DEGs in drought-treated M79, E1 and H1. Values are presented as Log_2_ (Fold Change). k: Scatter plots of expression values of 10 DEGs in drought-treated M79, E1 and H1. *X* and *Y* axes represent Log_2_ (Fold Change) obtained from RNA-seq and qRT-PCR experiments, respectively. **, Gene expression values for RNA-seq and qRT-PCR were significant (** *P* < 0.01).

To verify the reliability of our transcriptomic sequence data, we selected ten genes from all three lines for qRT-PCR, including genes encoding POD, No Apical Meristem, ATAF1/2, Cup-Shaped Cotyledon 2 (NAC) transcription factor, wax, lipid transfer proteins (LTPL78), Domain of unknown function (DUF538), expansin precursor, PsbP, Psb28, and two oxygen evolving enhancer proteins 3 (Table S2). Positive correlation coefficients between RNA-seq and qRT-PCR results was high and significant (*R*^2^ = 0.975, *P* < 0.01, Fig. 2), indicating that the transcriptome sequencing results were accurate and reliable.

### Comparative analysis of DEGs between M79 and its parental lines before and after drought stress

Comparative analysis of DEGs in non-stressed plants showed that M79 had 1359 and 648 genes up- and down-regulated, respectively, when compared to E1, and had 1496 and 1033, respectively, when compared to H1 (Fig. S2). To explore how these DEGs enhanced drought-resistance, a GO analysis was performed on highly DEGs identified between M79_CK and E1_CK, and between M79_CK and H1_CK. The DEGs found between M79 and E1 were mainly involved in ADP binding, RNA synthesis, post-translational modification, cell recognition, and carbohydrate metabolism (Table S3). In the comparison between M79 and H1, the DEGs were mainly related to protein kinase, iron binding, redox balance, and post-translational modification (Table S4).

**Figure 3:**
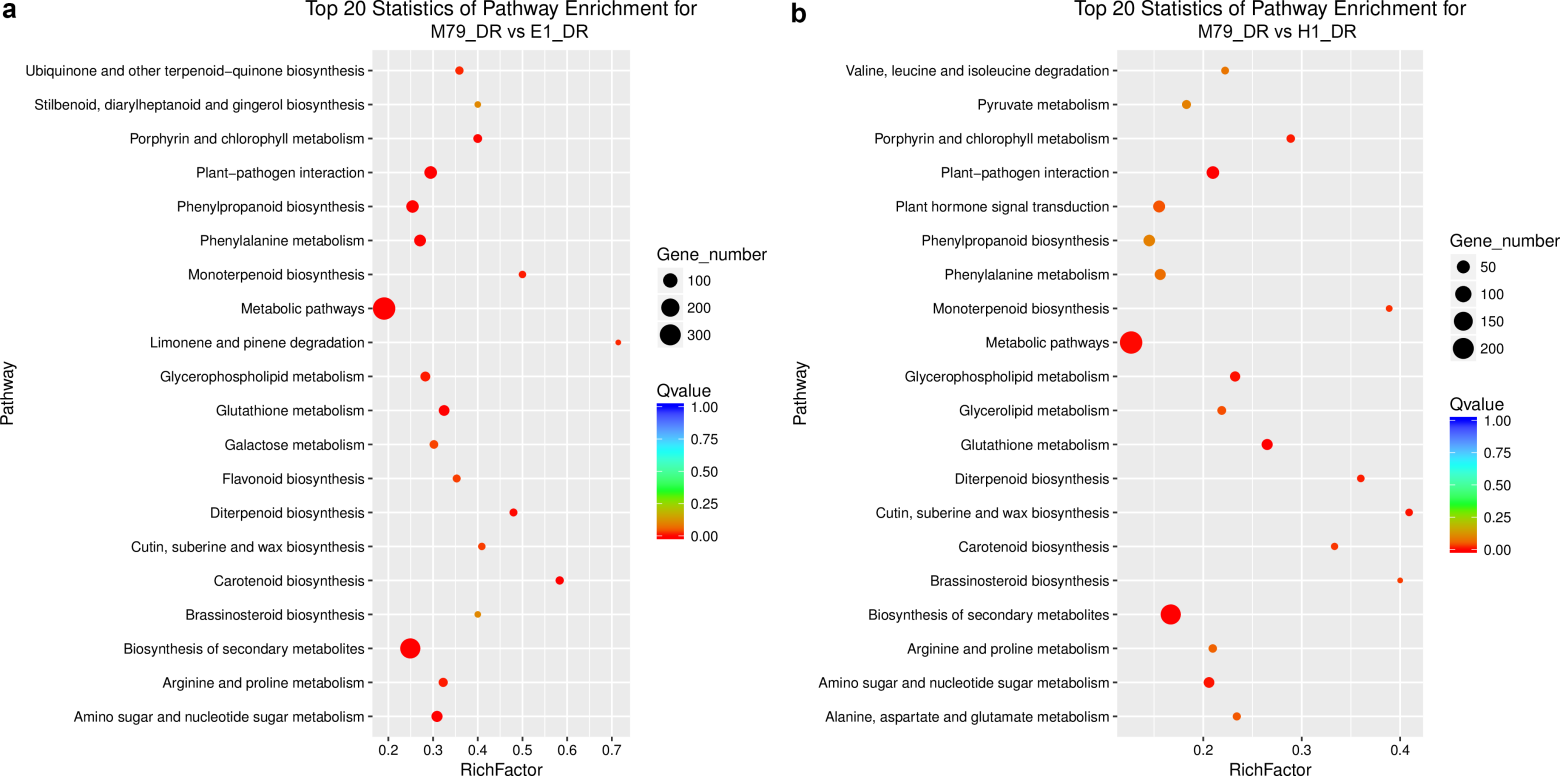
Scatter plots of enriched KEGG functional pathways in response to drought treatment. (A) M79_DR VS E1_DR; (B) M79_DR vs H1_DR. The “Rich Factor” shows the ratio of the number of the DEGs to the total gene number in certain pathways.

We analyzed the DEGs between M79_DR and E1_DR, and between M79_DR and H1_DR. 5,258 (2739 up-regulated, 2519 down-regulated) DEGs were identified between M79_DR and E1_DR, and 3594 (1795 up-regulated, 1799 down-regulated) DEGs were identified between M79_DR and H1_DR (Fig. S3). GO analysis showed that the DEGs between M79_DR and E1_DR, and between M79_DR and H1_DR were significantly enriched in protein kinase activity, ATP binding, iron ion binding, carbohydrate metabolism, redox balance, and post-translational modification (Tables S5 and S6). KEGG analysis on these DEGs found 18 pathways (*FDR* < 0.05) after the comparison between M79_DR and E1_DR, including metabolism of glutathione, phenylalanine, porphyrin, chlorophyll, arginine, and proline, and biosynthesis of phenylpropanoid, carotenoids, flavonoids, cuticle, suberin, and wax (Fig. 3A). Comparison between M79_DR and H1_DR identified 15 different pathways (*FDR* < 0.05) including metabolism of glutathione, porphyrin and chlorophyll, and biosynthesis of carotenoids, brassinosteroids, cuticle, suberin and wax, and plant hormone signaling (Fig. 3B).

### DEGs analysis of M79, E1 and H1 under drought stress

**Figure 4:**
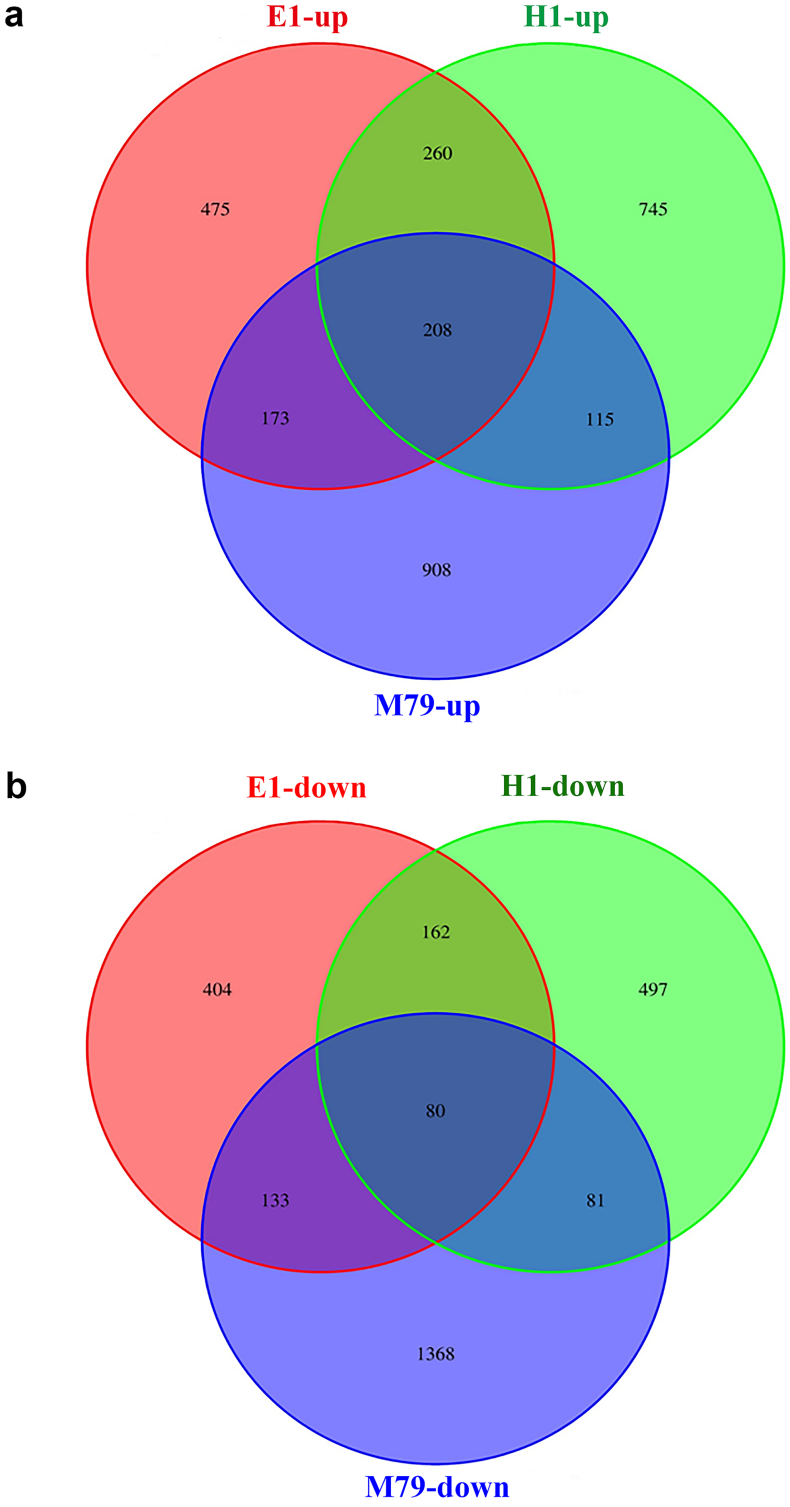
Venn diagrams of drought-responsive DEGs (both up- and down-regulated) in M79, E1 and H1. The DEGs were selected when FDR ≤ 0.05. (A) Up-regulated DEGs in three genotypes after drought treatment; (B) down-regulated DEGs in three genotypes after drought treatment.

Compared to untreated plants, 3066, 1895, and 2148 DEGs were identified after drought treatment in M79, E1 and H1, respectively, with 1404, 1116, and 1328 up-regulated genes and 1662, 779 and 820 down-regulated genes in corresponding genotypes. Among these DEGs, 288 (208 up-regulated and 80 down-regulated) genes were expressed in all three genotypes, accounting for 9.39%, 15.20%, and 13.41% of all DEGs in drought-treated M79, E1 and H1, respectively (Fig. 4). GO analysis showed that these genes were significantly enriched in carbohydrate metabolism, iron ion and heme binding, oxidoreductase, POD, protein kinase activity, and plasma membrane osmoregulation (Tables S7 and S8). Among them, genes known to be involved in drought tolerance included two POD precursors (Seita.5G174100 and Seita.8G015200), two late embryogenesis abundant proteins (LEAs) (Seita.1G015800 and Seita.5G021400), and two aquaporins (Seita.3G082100 and Seita.1G264900). Furthermore, genes involved in photosynthesis, such as one early light-induced protein (Seita.2G053800), one pheophorbide a oxygenase (PaO) (Seita.1G348100), and one senescence-inducible chloroplast stay-green protein 1 (SGR1) (Seita.2G285600) (Table S9) were also found. In addition, many transcription factors including basic leucine zipper (bZIP), NAC, v-myb avian c viral oncogene homolog (MYB) and early responsive to dehydration (ERD) family members, as well as genes related to calmodulin, protein kinase, and hormone (gibberellin (GA), ethylene (ETH)) biosynthesis and signaling (Table S9).

Further analysis of DEGs that were specifically expressed before and after drought stress detected 908, 475 and 745 up-regulated genes, and 1368, 404, and 497 down-regulated genes in M79, E1 and H1, respectively (Fig. 4). GO analysis showed that the drought-specific DEGs in M79 were significantly enriched in the GO-terms associated with PSII oxygen-evolving complex, carbohydrate metabolism, redox balance, and iron ion binding (Table S10). In contrast, the drought-specific DEGs were mainly enriched in GO-terms associated with iron ion binding, redox balance, and nucleic acid and transcription factor activity in E1 (Table S11), and with iron ion binding, protein kinase activity, and fatty acid synthesis in H1 (Table S12). KEGG analysis showed that the DEGs in drought-stressed M79 were mostly involved in pathways such as phenylalanine biosynthesis, plant hormone signaling, porphyrin, chlorophyll metabolism, cuticle and wax biosynthesis, and arginine and proline metabolism (Table S13). The pathways included phenylalanine biosynthesis, phenylalanine metabolism, plant hormone signaling, linoleic acid metabolism, and glycerophospholipid metabolism in E1 (Table S14), and phenylalanine biosynthesis, plant hormone signaling, and carotenoid biosynthesis in H1 (Table S15). These results suggested that those DEGs enabled the three genotypes to respond differently to drought stress.

### Expression and regulation of drought stress-responsive genes in M79

GO enrichment and KEGG pathway analysis indicated that these stress-induced DEGs in M79 were widely involved in signal transduction, transcriptional regulation, hormone signaling, redox regulation, osmotic regulation, photosynthesis, and other biological processes (Table S16).

Among the signal transduction-related genes that were differentially expressed in M79 after drought stress, 24 genes encode receptor kinases, of which 14 encoded wall-associated kinase receptor-like protein kinase (WAK-RLK) (eight up-regulated and six down-regulated), 27 genes encode protein kinases, of which 10 encode calcium/calmodulin dependent protein kinases (CAMK) (five up-regulated and five down-regulated), and six are Ca^2+^-related genes, including one up-regulated gene that encodes an EF hand family protein, and five genes encoding sodium/calcium exchanger (NCX) protein (three up-regulated and two down-regulated) (Table S16).

Ninety-six transcription factors were differentially expressed in M79 in response to drought stress, including 20 NAC, 19 APETALA2 (AP2), 14 transcription factors containing highly conserved protein domain (WRKY), seven bZIP, three ethylene response factor (ERF), and five dehydration-responsive element-binding (DREB) family transcription factors. These transcription factors played essential roles in M79 when it was exposed to drought stress (Table S16).

Many genes involved in phytohormone signaling were also responsible for drought tolerance in M79. We identified 17 DEGs that are related to auxin/indole-3-acetic acid (Aux/IAA) regulation, including nine of the *OsIAA* family (one up-regulated and eight down-regulated) and four belonging to the *OsSAUR* family (one up-regulated and three down-regulated). In addition, we identified one up-regulated cytokinin (CTK) dehydrogenase-related gene, ten DEGs involved in the GA regulation pathway, including six gibberellin 20 oxidases (four up-regulated and two down-regulated), three gibberellin 2-beta-dioxygenases (two up-regulated and one down-regulated), and one up-regulated gibberellin receptor *GID1L2*, and three DEGs related to ETH (two up-regulated and one down-regulated) (Table S16).

A total of 63 DEGs in M79 were identified to be involved in redox regulation, including genes encoding superoxide dismutase (SOD), glutathione peroxidase (GPx), oxidoreductase, POD, ascorbate peroxidase (APX), and lipoxygenase (LOX) (Table S16).

Forty-four DEGs related to osmotic regulation in M79. Among them, twelve DEGs were involved in proline metabolism, with ten up-regulated genes; seven DEGs were aquaporin genes with three up-regulated genes, and 12 DEGs belonging to ATP-binding cassette, subfamily G (ABCG) transporter family genes with six up-regulated genes (Table S16).

Forty-nine photosynthesis-related genes were differentially expressed in drought-treated M79 plants. These were involved in several photosynthetic metabolic pathways, including synthesis and degradation of chlorophyll, light energy absorption and transmission, PSII reaction center electron transfer, PSII reaction center electron transfer, and water oxidation. Among them, one PaO gene, one PsbP gene, one phytoene synthase gene, one scaffold protein in nitrogen fixation system (NifU) gene, and one ferrochelatase-2 gene were significantly up-regulated in M79 (Table S16). These genes maintained photosynthesis in M79 under drought.

### Co-regulation analysis of drought-responsive DEGs in M79

**Figure 5:**
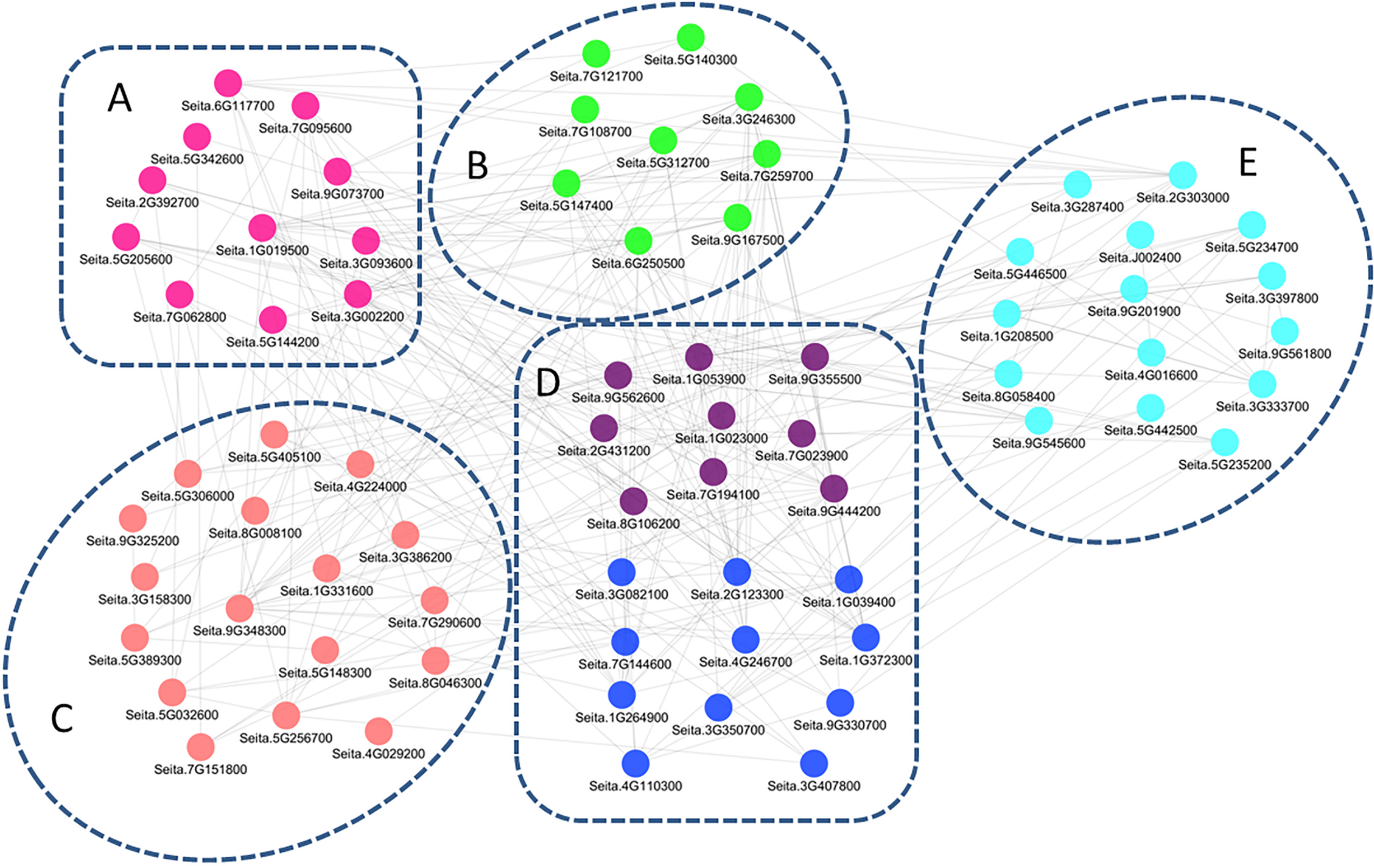
A regulatory network consisting of drought-responsive DEGs in M79. Pearson’s correlation coefficient |PCC| ≥ 0.93. The genes were categorized into five groups, with different colors representing different functional annotations: (A) signal transduction (pink), (B) phytohormones (green), (C) transcription factors (light pink), (D) redox (purple) and osmotic adjustment (dark blue), (E) photosynthesis (light blue).

The co-regulation study generated a regulatory network of 72 genes (Fig. 5), and the related genes were further divided into five groups. Genes in group A were mainly involved in signal transduction, including those encoding receptor kinases, Ca^2+^-related proteins, and protein kinases. Group B contained genes for GA, Aux/IAA, ETH, and CTK signaling. Group C consisted of transcriptional regulatory genes, including 4 bZIP, 1 DREB, and three WRKY family transcription factors. Genes in group D were drought-related, acting downstream of the molecular pathway responsible for drought tolerance in M79, including redox balance regulation genes (POD, GPx, and APX) and osmotic regulation-related genes (ion transporter, aquaporins, and proline synthesis-related genes). Group E contained photosynthesis-related genes, including two genes encoding oxygen evolving enhancer protein, three encoding PsbP proteins, and one encoding a ferrochelatase-2. The regulatory network was involved at all stages, and the drought response pathways in M79 may be essential for higher drought tolerance in M79 than its parental lines.

### Responses of drought-treated M79 in photosynthesis-related pathways

**Figure 6:**
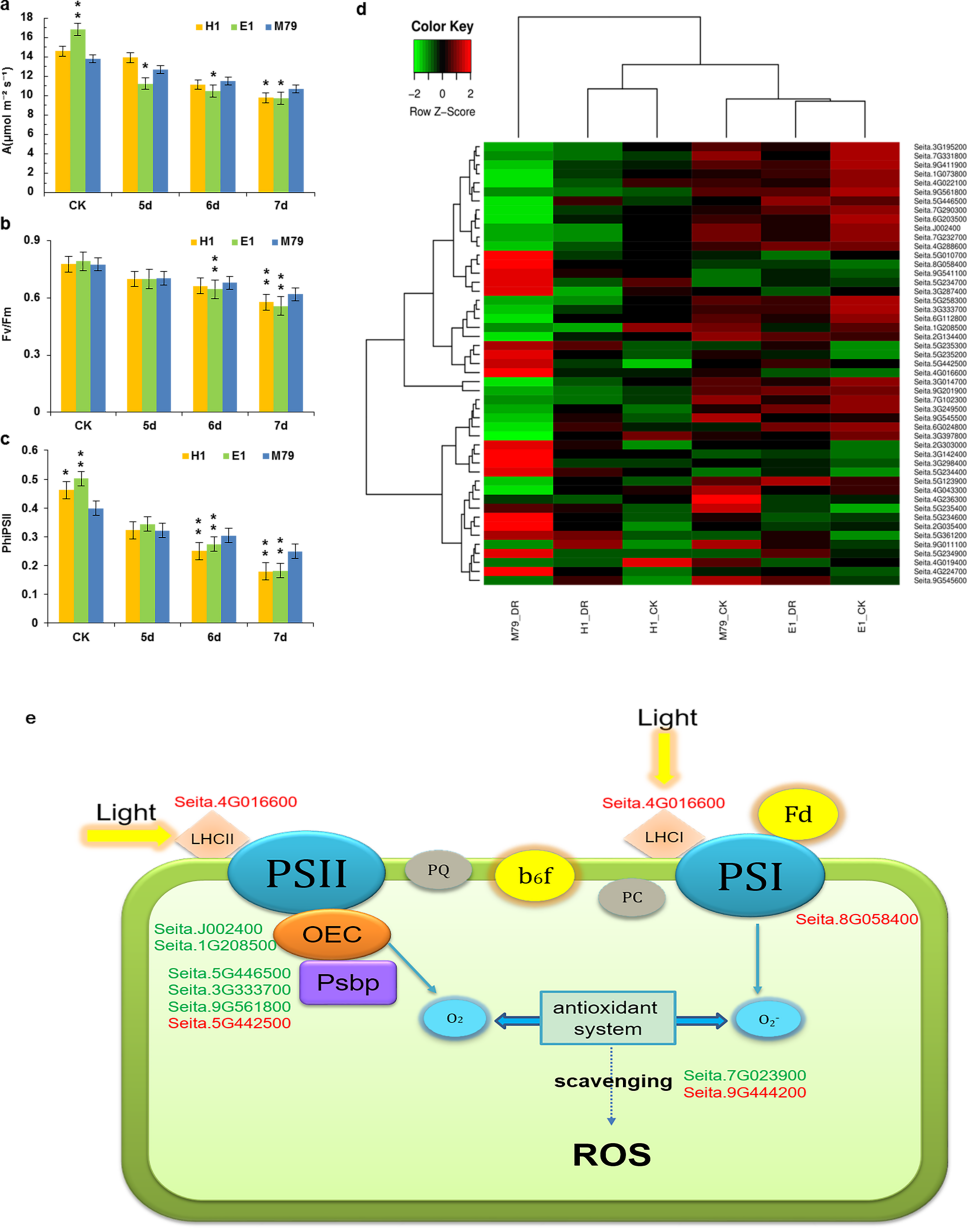
Photosynthetic analysis of drought-stressed M79, E1 and H1. (A) A (µmol m ^−2^ s ^−1^) of M79, E1 and H1 under normal conditions and 5–7 days after drought treatment; (B) Fv/Fm of M79, E1 and H1 under normal conditions and 5–7 days after drought treatment; (C) ΦPSII of M79, E1 and H1 under normal conditions and 5–7 days after drought treatment; (D) clustered heatmap showing photosynthetic DEGs in drought-stressed M79, E1 and H1 (E) photosynthetic pathways in drought-tolerant M79. Red and green indicate the up- and down-regulated DEGs, respectively, in response to drought stress. Data are presented as means ± SD (*n* = 5); *, significance levels in comparisons with M79 were determined by *t*-tests (* *P* < 0.05, ** *P* < 0.01).

As a C4 crop, the ability for millet to maintain photosynthesis under drought stress is an important indicator of drought resistance (Feng et al., 2015). The net photosynthetic rate of the three cultivars eventually decreased with prolonged drought stress. The net photosynthetic rate of E1 was the highest before drought treatment (16.82) in comparison with H1 (14.57) and M79 (13.77). After the 6th and 7th days of stresses, the net photosynthetic rate of M79 was the highest (11.52 and 10.67, respectively), reflecting the smallest decrease among the three cultivars in response to drought stress (Fig. 6A). Under drought stress, both Fv/Fm and ΦPSII showed a decreasing trend, and the parental lines E1 and H1 declined to a greater extent than M79 (Figs. 6B,  6C). These results demonstrated that the function of PSII was inhibited under drought stress, and that M79 maintained a relatively higher light energy utilization ratio than E1 and H1.

Functional annotation, GO enrichment and KEGG analysis identified 49 DEGs involved in the photosynthesis pathway responding to drought stress and showed higher expression levels in M79 than E1 and H1 (Fig. 6D). Further analysis of the photosynthetic pathway in M79 under drought stress showed that a gene encoding ferrochelatase-2 (Seita.4G016600) was up-regulated and involved in absorption and utilization of light energy during photosynthesis. Moreover, two genes encoding oxygen evolving enhancer protein (Seita.J002400 and Seita.1G208500) along with three PsbP genes (Seita.3G333700, Seita.9G561800 and Seita.5G442500) and one gene encoding the PSII reaction center Psb28 protein (Seita.5G446500) were involved in the breakage of water and oxygen release in the PSII reaction center. In addition, the NifU gene (Seita.8G058400) was up-regulated and involved in Fe-S cluster assembly in the PSII reaction center. Finally, two APX genes (Seita.7G023900 and Seita.9G444200) played a role in scavenging ROS (Fig. 6E). In addition, five genes (Seita.2G303000, Seita.5G234700, Seita.5G235200, Seita.6G024800 and Seita.9G545500) could be directly linked to the net photosynthetic rate and Fv/Fm based on the correlation analysis on physiological data and the expression of photosynthesis-related DEGs (Table S17). All of these genes regulated the photosynthetic pathway in M79 in response to drought stress.

## Discussion

### The drought responsive pathway and related genes in millet

Previous studies on drought-responsive pathways indicate that drought-inducible genes from different varieties of the same crop likely play a relatively conserved role in their regulatory networks (Yamaguchi-Shinozaki & Shinozaki, 2006). However, drought-induced genes codify not only proteins that directly protect the cell structure and related metabolic pathways, but also regulators with roles in stress signaling, and forming a set of elements responding to environmental stress (Caldana et al., 2011; Vermeirssen et al., 2014). Among them, WAK and CAMK are central in plant responses to abiotic stress (Zhang et al., 2005; Lv et al., 2014). Protein phosphatase 2C (PP2C) belongs to a group of phosphatases involved in ABA signaling, and is a negative regulator of ABA signaling (Zhang & Gan, 2012). Among the DEGs that we identified from three drought-treated cultivars, four genes encoding CAMK kinases were up-regulated, three genes encoding WAK receptor kinases were down-regulated, and seven PP2C genes were up-regulated in all three cultivars (Table S9). Aux/IAA, ETH, and GA are phytohormones playing an important part in maintaining normal plant growth and development, and in reacting to abiotic stresses (Arraes et al., 2015; Gaion et al., 2017; Yu et al., 2017). Our study detected one down-regulated gene encoding for Aux/IAA, two ETH-encoding genes (one up-regulated and one down-regulated) and three GA-encoding genes (one up-regulated and two down-regulated) in all three drought-treated cultivars (Table S9). Transcription factor families such as NAC, MYB, bZIP, and basic helix-loop-helix (bHLH) also have a role in plant responses to both biotic and abiotic stresses (Katiyar et al., 2012; Puranik et al., 2013; Yong et al., 2014). Twelve DEGs belonging to these families were expressed in all three cultivars. They include five up-regulated genes encoding NACs, five genes encoding MYBs (three up-regulated and two down-regulated), an up-regulated gene encoding a bZIP and an up-regulated gene encoding a bHLH transcription factor (Table S9). Many protective proteins are also considered essential for protecting plants from damage caused by drought stress. For example, drought stress induces the expression of LEAs, which reduce water loss in plant cells and increase their WUE under adverse conditions (Wang et al., 2014). Genes encoding for protective proteins that were up-regulated in all three cultivars in our study included three coding for universal stress proteins (USP) and two for LEAs (Table S9).

A variety of intrinsic membrane proteins protect plant cells from abiotic stress by regulating the permeability of the plasma membrane (Kasim et al., 2015). Here, we detected two genes encoding aquaporins, one up-regulated and one down-regulated (Table S9). Plant cuticle is a hydrophobic protective layer that prevents water loss and protects plants from abiotic stress, such as those created from exposition to high temperature, drought, and salt (Ma et al., 2015). We detected both up-regulated genes encoding waxs in our experiments (Table S9). The antioxidant defense system in plants under drought stress is composed of ROS scavenging enzymes. Among them, CAT, SOD, APX, and GPx are essential to remove ROS and act synergistically to counteract oxidative damage caused by drought stress (Adriano et al., 2015). Our results indicated that one gene encoding POD was up-regulated and one down-regulated (Table S9). In addition, LTPs play an important role in response to biotic and abiotic stresses. We detected seven LTPL-coding genes up-regulated in all three cultivars, suggesting a positive role for these proteins in the drought defensive pathway (Safi et al., 2015 Table S9). All DEGs identified in drought-treated M79, E1 and H1 were involved in drought-defensive processes, and the transcription of many of them was up-regulated, suggesting that these genes play a positive regulatory role in drought response.

### Molecular basis for better drought tolerance in M79 than its parental lines

RNA-seq analysis identified 5258 DEGs between M79 and E1, and 3594 between M79 and H1, indicating that the drought-tolerant cultivar M79 and its parental lines had different transcriptional profiles (Fig. S3). GO analysis of these DEGs showed that they were highly enriched in GO-terms such as membrane, protein kinase activity, transferase activity, carbohydrate metabolism, iron ion binding, ATP binding, heme binding, oxidoreductase activity, phosphorylation, and protein modification (Tables S5, S6). Among them, metal ion binding, electron-carrier activity, and expression of genes related to oxidoreductase synthesis increase the ability of plants to resist drought and high temperature (Rizhsky et al., 2004). In addition, phosphorylation is involved in stress responses in plants. In Arabidopsis, the SUCROSE NONFERMENTING1 (SNF1) kinase homologs 10 and 11 play an essential role in stress responses (Chen & Hoehenwarter, 2015).

KEGG analysis found 18 and 15 significant pathways that differentiate between M79_DR and E1_DR (*FDR* < 0.05), and between M79_DR and H1_DR (*FDR* < 0.05). These pathways include biosynthesis of secondary metabolites, plant-pathogen interaction, metabolic pathways, carotenoid biosynthesis, glutathione metabolism, amino sugar and nucleotide sugar metabolism, phenylalanine metabolism, phenylpropanoid biosynthesis, porphyrin and chlorophyll metabolism, diterpenoid biosynthesis, monoterpenoid biosynthesis, arginine and proline metabolism, glycerophospholipid metabolism, ubiquinone and other terpenoid-quinone biosynthesis, limonene and pinene degradation, flavonoid biosynthesis, cutin, suberine and wax biosynthesis, galactose metabolism, brassinosteroid biosynthesis, glycerolipid metabolism, plant hormone signal transduction, and alanine, aspartate and glutamate metabolism (Fig. 3). Among them, glutathione metabolism can reduce and eliminate oxidative damage caused by ROS, and it plays an important role in maintaining redox balance (Hicks et al., 2007). As an important osmotic regulator in plants, proline helps to maintain osmotic pressure, and it stabilizes proteins and cellular structures under drought stress (Vendruscolo et al., 2007). Phenylalanine and flavonoids also play an important role in adapting plants to stress and overcoming stress damage (Hernández, Alegre & Munné-Bosch, 2006; Babst et al., 2014; Pan et al., 2018). The cuticle and wax contribute in protecting plants from biotic and abiotic stresses, and in maintaining plant morphology (Pollard et al., 2008). Using high-throughput Illumina RNA-seq, Wang et al. (2016a) identified and quantified DEGs related to flavonoid biosynthesis in drought-stressed plants. Biosynthesis of brassinosteroids, monoterpenoids, porphyrins, chlorophyll, ubiquinone, and other terpenoid-quinones also is instrumental in adapting plants to drought stress and overcoming stress damage (Ksouri et al., 2016; Hajrah et al., 2017; Miao et al., 2017). In addition, KEGG pathway analysis indicated a significant enrichment in amino sugar and nucleotide sugar metabolism, and limonene and pinene degradation in response to abiotic stress (Singh et al., 2017; Zhang et al., 2017).

When challenged with drought stress, the transcriptional profile and ability to tolerate drought stress in M79 were significantly different from those of both parental lines. Crossbreeding is a means to promote recombination of parental genes, and is also a prerequisite for breeding superior offspring, that not only inherit desired traits from both parental lines, but also can exhibit novel F_1_ phenotypes, heterosis and modified gene expression (Shivaprasad et al., 2012; Bell et al., 2013). However, the role of heterosis in defense mechanism of abiotic stress is still poorly understood (Dong et al., 2006; Korn et al., 2008; Singh, Sharma & Singh, 2010; Miller et al., 2015). Korn et al. (2008) showed that there is a strikingly strong correlation between heterosis, freezing tolerance, and flavonol content. The heterotic vigor for SOD, POD and CAT suggests an improvement of stress tolerance level in hybrids compared to the parental lines (Singh, Sharma & Singh, 2010). Miller et al. (2015) found that the levels of stress-responsive gene expression in parental lines could be used to predict biomass heterosis in hybrids. We therefore hypothesized that the reason for the higher drought stress tolerance in M79 than its parental lines was due to heterosis. However, this hypothesis needs further examination.

### Molecular co-regulatory network for drought tolerance in millet

The millet genome has a complex molecular regulatory network to cope with drought, which can activate specific cell signaling pathways and induce transcriptional regulation that leads to enhanced cellular responses, increased expression of antioxidant-related genes, and accumulation of soluble substances (Lata, Sahu & Prasad, 2010). We analyzed DEGs in M79 before and after drought stress. Genes that are widely involved in biological processes such as signal transduction, hormonal signaling, transcriptional regulation, redox regulation, osmotic adjustment, and photosynthesis formed a drought-tolerance regulatory network (Table S16).

Many signal transduction-related genes were up-regulated after drought treatment, including five genes encoding NCX proteins (Table S16). This gene family is involved in Ca^2+^ signaling, and dehydration induces the expression of *OsNCX3*, *OsNCX10*, and *OsNCX15* in rice (Singh et al., 2015). Therefore, NCXs in M79 may be involved in signal transduction during drought stress to activate the expression of downstream genes. In addition, we found 14 DEGs (eight up-regulated) encoding WAKs, and 10 (five up-regulated) encoding CAMKs (Table S16). WAKs belong to a receptor-like kinase gene family, and respond to abiotic stress acting on the signal transduction between the cell wall and cytoplasm (Zhang et al., 2005), whereas CAMKs have roles in Ca^2+^ signaling and protein phosphorylation (Chen, Zhang & Cheng, 2017). These up-regulated genes positively regulated drought-related signaling pathways in M79, contributing to drought stress tolerance with a rapid activation of downstream gene expression.

ETH, CTK, Aux/IAA, and GA are all involved in plant response to abiotic stresses (Werner & Schmülling, 2009; De Diego et al., 2013; Colebrook et al., 2014; Zwack & Rashotte, 2015; Yang et al., 2015; Yu et al., 2017). CTK and Aux/IAA negatively regulate ABA-induced stomatal closure (Werner & Schmülling, 2009; De Diego et al., 2013). Colebrook et al. (2014) showed that decreased GA content and transcriptional alteration of related genes inhibit plant growth and development under various abiotic stress conditions. Yang et al. (2015) demonstrated that ETH is involved in salt stress-related responses in rice, and that it plays a vital role in regulating biotic and abiotic stress responses. In this study, we found two, one, three, and seven up-regulated genes involved in ETH, CTK, Aux/IAA, and GA pathways, respectively, in M79 following drought stress (Table S16). These genes maintain growth and development in plants under drought stress by regulating hormone balance.

Transcription factors play a central role in biotic and abiotic stress responses, and in the regulation of various biological processes. AP2/DREB, WRKYs, ERF, bHLHs, bZIP and NAC transcription factor families are involved in transcriptional regulation in response to stress (Lata et al., 2014; Li et al., 2014; Muthamilarasan et al., 2015). Expression analysis of the millet AP2/ERF genes *SiAP2/ERF-069, SiAP2/ERF-103,* and *SiAP2/ERF-120* showed that they were all up-regulated under drought stress and therefore may play a positive role in this process (Lata et al., 2014). *SiARDP* belongs to the DREB family of transcription factors and is one of the target genes of *SiAREB*; it participates in the ABA-dependent signaling pathway. Overexpression of *SiARDP* improves drought resistance in millet (Li et al., 2014). In addition, Muthamilarasan et al. (2015) showed that the *SiWRKY* genes *SiWRKY066* and *SiWRKY08* give an essential contribution in response to abiotic stress. In this study, multiple members of these families were differentially expressed after drought stress, including 20 NACs, 19 AP2s, 14 WRKYs, seven bZIPs, three ERFs, and five DREBs, indicating their involvement in the responses to drought stress (Table S16). In addition, Komivi, Diaga & Ndiaga (2016) showed that 90% of heat shock factors (HSFs) respond to drought stress in sesame seedlings, and that two HSF transcription factors are significantly up-regulated after drought stress, suggesting that these genes might contribute to this process (Table S16).

Upon drought stress, gene expression is pivotal in protecting plants from oxidative damage. For example, SOD converts superoxide into the less toxic H_2_O_2_, which is then reduced to H_2_O by POD, APX, and GPx (Alscher, Erturk & Heath, 2002; Fecht-Christoffers et al., 2006; Islam, Manna & Reddy, 2015). In our study, drought stress up-regulated 10 genes encoding PODs, two genes encoding GPx, and one gene encoding APX in M79. These genes function as positive regulators removing ROS and maintaining a redox balance (Table S16).

Our study detected a large number of genes related to osmoregulation in M79 after drought stress, including 12 (10 up-regulated) involved in proline metabolism, 12 (six up-regulated) ABCG transporters, seven (three up-regulated) aquaporins, and two (both up-regulated) ATP-binding cassette (ABC) transporters (Table S16). Proline is an important macromolecule serving as an osmotic regulator in stress-defensive responses, since its accumulation can relieve damage caused by osmotic stress under drought (An et al., 2013). ABC transporters utilize ATP hydrolysis to transport osmotic regulators such as amino acids, peptides, carbohydrates, lipids, hormones and metal ions (Jeong et al., 2014). Aquaporin proteins assist the plant to combat abiotic stress by regulating the permeability of the plasma membrane (Kasim et al., 2015). ABCG transporters help to biosynthesize protective cuticles and wax, transporting lipids or to regulate phytohormone homeostasis transporting indole butyric acid and ABA (Yadav et al., 2014). Expression of the above genes can regulate the osmotic potential of M79 cells under drought stress to reduce injury.

Co-regulation analysis of these DEGs in M79 revealed a regulatory network consisting of 72 genes, which might contribute to the excellent drought resistance of M79. This system includes signal perception and transduction, hormone signaling pathways, transcriptional regulatory factors, and downstream functional genes (including ROS removal factors, ion transporters and osmotic regulators) (Fig. 5). Although the results of our co-regulation analysis remain to be further verified, the co-regulation network provides an important theoretical basis to propose a model for the molecular mechanisms of drought tolerance in millet.

### Maintenance of a high photosynthetic rate is an important indicator of drought tolerance in crop plants

Photosynthesis is the basic metabolism regulating crop growth and final yield. The maintenance of photosynthetic rates under drought stress is essential for drought tolerance in crops (Galmés, Medrano & Flexas, 2007; Chaves, Flexas & Pinheiro, 2009). We found that photosynthetic rate and light energy utilization in E1 and H1 was significantly lower than in M79 after drought stress, suggesting that M79 can maintain higher photosynthesis under drought (Figs. 6A– 6C). Therefore, photosynthetic rate under drought stress is not only related to photosynthetic capacity, but also to drought tolerance (Zhang, Li & Xiao, 2016).

Photosynthesis in chloroplasts converts light into chemical energy that is used for plant growth and development. Under drought stress, O_2_ produced in chloroplasts can receive electrons from the photosynthetic electron transport chain to become O^−2^, which can cause oxidative damage to photosynthetic pigments and the plasma membranes (Gill & Tuteja, 2010; Exposito-Rodriguez et al., 2017). Previous studies showed that osmoregulation and antioxidant capacity of plants contribute to maintaining photosynthetic capacity (Ramachandra Reddy, Chaitanya & Vivekanandan, 2004). Our co-regulatory analysis showed that osmotic regulation and antioxidation played a vital role in the management of drought tolerance in M79 (Fig. 5; Table S16), explaining why M79 was able to maintain a high net photosynthetic rate under drought stress.

Our results identified 49 photosynthesis-related DEGs in drought-treated M79. Among them, 11 genes were involved in the photosynthetic pathway, including one gene encoding ferrochelatase-2 (Seita.4G016600), two genes encoding oxygen evolving enhancer proteins (Seita.J002400 and Seita.1G208500), three PsbP-encoding genes (Seita.3G333700, Seita.9G561800 and Seita.5G442500), one gene encoding PSII reaction center Psb28 protein (Seita.5G446500), one NifU-encoding gene (Seita.8G058400), and two APX-encoding genes (Seita.7G023900 and Seita.9G444200) (Fig. 6E). Previous studies showed that ferrochelatase is related to the absorption of light by the light-harvesting complex (LHC) proteins (Suzuki et al., 2002; Espinas et al., 2016). PsbP and Psb28, two subunits of the PSII reaction center, are involved in the water photolysis and oxygen release during photosynthesis (Mabbitt, Wilbanks & Eaton-Rye, 2014). NifU plays an important role in the synthesis and assembly of the Fe-S cluster in PSI (Yabe et al., 2004). Under drought stress, electron transport and photosynthetic phosphorylation in chloroplasts produce a large amount of ROS, while APX effectively removes them and reduces oxidative damage to plants (Jiang et al., 2016; Exposito-Rodriguez et al., 2017). Up-regulation of these genes enables M79 to maintain a relatively high level of photosynthesis under drought stress, and to resist the damage caused by drought. Through the analysis of the photosynthetic pathway in drought-stressed M79, we provide an initial insight to the molecular mechanism of drought resistance.

## Conclusions

After the exposure of the F_1_ hybrid M79 and its parental lines (E1 and H1) to drought stress treatment, we demonstrated that M79 had higher photosynthetic energy conversion efficiency and better tolerance to drought stress when compared to its parental lines. Transcriptomic study suggested that DEGs in M79 contributed to the formation of a regulatory network involving multiple biological processes and pathways, including photosynthesis, signal transduction, transcriptional regulation, redox regulation, hormonal signaling, and osmotic regulation. We also demonstrated that, upon drought treatment, some photosynthesis-related DEGs were highly expressed in M79 compared to its parental lines. Finally, this study revealed critical molecular pathways, such as photosynthesis, involved in the responses to drought stress in M79, and provided abundant genetic information for further study of the underlying mechanism.

##  Supplemental Information

10.7717/peerj.4752/supp-1 Table S1 Descriptive statistical analysis of RNA-seq of 18 samples

10.7717/peerj.4752/supp-2 Table S2 Primers used for validating 10 DEGs by qRT-PCR

10.7717/peerj.4752/supp-3 Table S3 GO enrichment analysis of DEGs in M79 and E1 under normal conditions

10.7717/peerj.4752/supp-4 Table S4 GO enrichment analysis of DEGs in M79 and H1 under normal conditions

10.7717/peerj.4752/supp-5 Table S5 GO enrichment analysis of DEGs in M79 and E1 after drought treatment

10.7717/peerj.4752/supp-6 Table S6 GO enrichment analysis of DEGs in M79 and H1 after drought treatment

10.7717/peerj.4752/supp-7 Table S7 GO enrichment analysis of DEGs that were up-regulated in all three drought-treated cultivars

10.7717/peerj.4752/supp-8 Table S8 GO enrichment analysis of DEGs that were down-regulated in all three drought-treated cultivars

10.7717/peerj.4752/supp-9 Table S9 DEGs that were expressed in all three drought-treated cultivars

10.7717/peerj.4752/supp-10 Table S10 GO enrichment analysis of M79-specific DEGs after drought stress

10.7717/peerj.4752/supp-11 Table S11 GO enrichment analysis of E1-specific DEGs after drought stress

10.7717/peerj.4752/supp-12 Table S12 GO enrichment analysis of H1-specific DEGs after drought stress

10.7717/peerj.4752/supp-13 Table S13 KEGG analysis of M79-specific DEGs after drought stress

10.7717/peerj.4752/supp-14 Table S14 KEGG analysis of E1-specific DEGs after drought stress

10.7717/peerj.4752/supp-15 Table S15 KEGG analysis of H1-specific DEGs after drought stress

10.7717/peerj.4752/supp-16 Table S16 Functional classification of drought resistance-related DEGs in M79

10.7717/peerj.4752/supp-17 Table S17 Pearson correlation coefficients of physiological data and the expression data of photosynthesis-related DEGs

10.7717/peerj.4752/supp-18 Figure S1 Distribution of gene expression (FPKM) in 18 samples after drought treatment (A) Density distribution; (B) box-plot.

10.7717/peerj.4752/supp-19 Figure S2 Venn diagrams of DEGs (up-regulated and down-regulated) of M79, E1 and H1 before drought treatment

10.7717/peerj.4752/supp-20 Figure S3 Venn diagrams of DEGs (up-regulated and down-regulated) of M79, E1 and H1 after drought treatment

10.7717/peerj.4752/supp-21 Supplemental Information 1 Raw data

## Additional Information and Declarations

### Data Availability

The following information was supplied regarding data availability: Both raw data and code are included in the Supplemental Files.

## References

[ref-1] Adriano S, Antonio S, Maria N, Antonella V (2015). Ascorbate peroxidase and catalase activities and their genetic regulation in plants subjected to drought and salinity stresses. International Journal of Molecular Sciences.

[ref-2] Alscher RG, Erturk N, Heath LS (2002). Role of superoxide dismutases (SODs) in controlling oxidative stress in plants. Journal of Experimental Botany.

[ref-3] Ambavaram MMR, Basu S, Krishnan A, Ramegowda V, Batlang U, Rahman L, Baisakh N, Pereira A (2014). Coordinated regulation of photosynthesis in rice increases yield and tolerance to environmental stress. Nature Communications.

[ref-4] An Y, Zhang M, Liu G, Han R, Liang Z (2013). Proline accumulation in leaves of *Periploca sepium* via both biosynthesis up-regulation and transport during recovery from severe drought. PLOS ONE.

[ref-5] Arraes FB, Beneventi MA, Lisei de Sa ME, Paixao JF, Albuquerque EV, Marin SR, Purgatto E, Nepomuceno AL, Grossi-de Sa MF (2015). Implications of ethylene biosynthesis and signaling in soybean drought stress tolerance. BMC Plant Biology.

[ref-6] Babst BA, Chen HY, Wang HQ, Payyavula RS, Thomas TP, Harding SA, Tsai CJ (2014). Stress-responsive hydroxycinnamate glycosyltransferase modulates phenylpropanoid metabolism in *Populus*. Journal of Experimental Botany.

[ref-7] Bell GD, Kane NC, Rieseberg LH, Adams KL (2013). RNA-seq analysis of allele-specific expression, hybrid effects, and regulatory divergence in hybrids compared with their parents from natural populations. Genome Biology and Evolution.

[ref-8] Bennetzen JL, Schmutz J, Wang H, Percifield R, Hawkins J, Pontaroli AC, Estep M, Feng L, Vaughn JN, Grimwood J, Jenkins J, Barry K, Lindquist E, Hellsten U, Deshpande S, Wang X, Wu X, Mitros T, Triplett J, Yang X, Ye CY, Mauro-Herrera M, Wang L, Li P, Sharma M, Sharma R, Ronald PC, Panaud O, Kellogg EA, Brutnell TP, Doust AN, Tuskan GA, Rokhsar D, Devos KM (2012). Reference genome sequence of the model plant Setaria. Nature Biotechnology.

[ref-9] Bonnecarrère V, Borsani O, Díaz P, Capdevielle F, Blanco P, Monza J (2011). Response to photoxidative stress induced by cold in *japonica* rice is genotype dependent. Plant Science.

[ref-10] Boyle EI, Weng S, Gollub J, Jin H, Botstein D, Cherry JM, Sherlock G (2004). Go::TermFinder-open source software for accessing Gene Ontology information and finding significantly enriched Gene Ontology terms associated with a list of genes. Bioinformatics.

[ref-11] Caldana C, Degenkolbe T, Cuadros-Inostroza A, Klie S, Sulpice R, Leisse A, Steinhauser D, Fernie AR, Willmitzer L, Hannah MA (2011). High-density kinetic analysis of the metabolomic and transcriptomic response of Arabidopsis to eight environmental conditions. Plant Journal.

[ref-12] Camilios-Neto D, Bonato P, Wassem R, Tadra-Sfeir MZ, Brusamarello-Santos LCC, Valdameri G, Donatti L, Faoro H, Weiss VA, Chubatsu LS, Pedrosa FO, Souza EM (2014). Dual RNA-seq transcriptional analysis of wheat roots colonized by *Azospirillum brasilense* reveals up-regulation of nutrient acquisition and cell cycle genes. BMC Genomics.

[ref-13] Cao WH, Liu J, He XJ, Mu RL, Zhou HL, Chen SY, Zhang JS (2007). Modulation of ethylene responses affects plant salt-stress responses. Plant Physiology.

[ref-14] Chaves MM, Flexas J, Pinheiro C (2009). Photosynthesis under drought and salt stress: regulation mechanisms from whole plant to cell. Annals of Botany.

[ref-15] Chen Y, Hoehenwarter W (2015). Changes in the phosphoproteome and metabolome link early signaling events to rearrangement of photosynthesis and central metabolism in salinity and oxidative stress response in Arabidopsis. Plant Physiology.

[ref-16] Chen F, Zhang L, Cheng ZM (2017). The calmodulin fused kinase novel gene family is the major system in plants converting Ca^2+^ signals to protein phosphorylation responses. Scientific Reports.

[ref-17] Colebrook EH, Thomas SG, Phillips AL, Hedden P (2014). The role of gibberellin signaling in plant responses to abiotic stress. Journal of Experimental Biology.

[ref-18] Cui Y, Wang M, Zhou H, Li M, Huang L, Yin X, Zhao G, Lin F, Xia X, Xu G (2016). OsSGL, a novel DUF1645 domain-containing protein, confers enhanced drought tolerance in transgenic rice and *Arabidopsis*. Frontiers in Plant Science.

[ref-19] De Diego N, Rodríguez JL, Dodd IC, Pérez-Alfocea F, Moncaleán P, Lacuesta M (2013). Immunolocalization of IAA and ABA in roots and needles of radiata pine (*Pinus radiata*) during drought and rewatering. Tree Physiology.

[ref-20] Dong J, Wu F, Jin Z, Huang Y (2006). Heterosis for yield and some physiological traits in hybrid cotton cikangza 1. Euphytica.

[ref-21] Espinas NA, Kobayashi K, Sato Y, Mochizuki N, Takahashi K, Tanaka R, Masuda T (2016). Allocation of heme is differentially regulated by ferrochelatase isoforms in *Arabidopsis* cells. Frontiers in Plant Science.

[ref-22] Exposito-Rodriguez M, Laissue PP, Yvon-Durocher G, Smirnoff N, Mullineaux PM (2017). Photosynthesis-dependent H_2_O_2_ transfer from chloroplasts to nuclei provides a high-light signalling mechanism. Nature Communications.

[ref-23] Fecht-Christoffers MM, Führs H, Braun HP, Horst WJ (2006). The role of hydrogen peroxide-producing and hydrogen peroxide-consuming peroxidases in the leaf apoplast of cowpea in manganese tolerance. Plant Physiology.

[ref-24] Feng ZJ, He GH, Zheng WJ, Lu PP, Chen M, Gong YM, Ma YZ, Xu ZS (2015). Foxtail millet NF-Y families: genome-wide survey and evolution analyses identified two functional genes important in abiotic stresses. Frontiers in Plant Science.

[ref-25] Fracasso A, Trindade LM, Amaducci S (2016). Drought stress tolerance strategies revealed by RNA-Seq in two sorghum genotypes with contrasting WUE. BMC Plant Biology.

[ref-26] Gaion LA, Monteiro CC, Cruz FJR, Rossatto DR, López-Díaz I, Carrera E, Lima JE, Peres LEP, Carvalho RF (2017). Constitutive gibberellin response in grafted tomato modulates root-to-shoot signaling under drought stress. Journal of Plant Physiology.

[ref-27] Galmés J, Medrano H, Flexas J (2007). Photosynthetic limitations in response to water stress and recovery in Mediterranean plants with different growth forms. New Phytologist.

[ref-28] Gill SS, Tuteja N (2010). Reactive oxygen species and antioxidant machinery in abiotic stress tolerance in crop plants. Plant Physiology and Biochemistry.

[ref-29] Gollan PJ, Tikkanen M, Aro EM (2015). Photosynthetic light reactions: integral to chloroplast retrograde signalling. Current Opinion in Plant Biology.

[ref-30] Gürel F, Öztürk ZN, Uçarlhi C, Rosellini D (2016). Barley genes as tools to confer abiotic stress tolerance in crops. Frontiers in Plant Science.

[ref-31] Hajrah NH, Obaid AY, Atef A, Ramadan AM, Arasappan D, Nelson CA, Edris S, Mutwakil MZ, Alhebshi A, Gadalla NO, Makki RM, Al-Kordy MA, El-Domyati FM, Sabir JSM, Khiyami MA, Hall N, Bahieldin A, Jansen RK (2017). Transcriptomic analysis of salt stress responsive genes in Rhazya stricta. PLOS ONE.

[ref-32] He GH, Xu YJ, Wang XY, Liu MJ, Li SP, Chen M, Ma YZ, Xu ZS (2016). Drought-responsive WRKY transcription factor genes *TaWRKY1* and *TaWRKY33* from wheat confer drought and/or heat resistance in *Arabidopsis*. BMC Plant Biology.

[ref-33] Hernández I, Alegre L, Munné-Bosch S (2006). Enhanced oxidation of flavan-3-ols and proanthocyanidin accumulation in water-stressed tea plants. Phytochemistry.

[ref-34] Hicks LM, Cahoon RE, Bonner ER, Rivard RS, Sheffield J, Jez JM (2007). Thiol-based regulation of redox-active glutamate-cysteine ligase from *Arabidopsis thaliana*. The Plant Cell.

[ref-35] Hu H, Xiong L (2014). Genetic engineering and breeding of drought-resistant crops. Annual Review of Plant Biology.

[ref-36] Islam T, Manna M, Reddy MK (2015). Glutathione peroxidase of *Pennisetum glaucum* (PgGPx) is a functional Cd^2+^ dependent peroxiredoxin that enhances tolerance against salinity and drought stress. PLOS ONE.

[ref-37] Jeong CB, Kim BM, Lee JS, Rhee JS (2014). Genome-wide identification of whole ATP-binding cassette (ABC) transporters in the intertidal copepod *Tigriopus japonicus*. BMC Genomics.

[ref-38] Jiang G, Yin D, Zhao J, Chen H, Guo L, Zhu L, Zhai W (2016). The rice thylakoid membrane-bound ascorbate peroxidase *OsAPX8* functions in tolerance to bacterial blight. Scientific Reports.

[ref-39] Kanehisa M, Araki M, Goto S, Hattori M, Hirakawa M, Itoh M, Katayama T, Kawashima S, Okuda S, Tokimatsu T, Yamanishi Y (2008). KEGG for linking genomes to life and the environment. Nucleic Acids Research.

[ref-40] Kasim K, Pallavi A, Arti S, Vidhu AS (2015). Heterologous expression of two Jatropha aquaporins imparts drought and salt tolerance and improves seed viability in transgenic *Arabidopsis thaliana*. PLOS ONE.

[ref-41] Katiyar A, Smita S, Lenka SK, Rajwanshi R, Chinnusamy V, Bansal KC (2012). Genome-wide classification and expression analysis of *MYB*, transcription factor families in rice and *arabidopsis*. BMC Genomics.

[ref-42] Kim D, Pertea G, Trapnell C, Pimentel H, Kelley R, Salzberg SL (2013). Tophat2: accurate alignment of transcriptomes in the presence of insertions, deletions and gene fusions. Genome Biology.

[ref-43] Komivi D, Diaga D, Ndiaga C (2016). Genome-wide investigation of Hsf genes in sesame reveals their segmental duplication expansion and their active role in drought stress response. Frontiers in Plant Science.

[ref-44] Korn M, Peterek S, Mock HP, Heyer AG, Hincha DK (2008). Heterosis in the freezing tolerance, and sugar and flavonoid contents of crosses between *Arabidopsis thaliana* accessions of widely varying freezing tolerance. Plant Cell and Environment.

[ref-45] Ksouri N, Jiménez S, Wells CE, Contreras-Moreira B, Gogorcena Y (2016). Transcriptional responses in root and leaf of *Prunus persica* under drought stress using RNA sequencing. Frontiers in Plant Science.

[ref-46] Lata C, Gupta S, Prasad M (2013). Foxtail millet: a model crop for genetic and genomic studies in bioenergy grasses. Critical Reviews in Biotechnology.

[ref-47] Lata C, Mishra AK, Muthamilarasan M, Bonthala VS, Khan Y, Prasad M (2014). Genome-wide investigation and expression profiling of AP2/ERF transcription factor superfamily in foxtail millet (*Setaria italica* L.). PLOS ONE.

[ref-48] Lata C, Sahu PP, Prasad M (2010). Comparative transcriptome analysis of differentially expressed genes in foxtail millet (*Setaria italica* L.) during dehydration stress. Biochemical and Biophysical Research Communications.

[ref-49] Li P, Brutnell TP (2011). *Setaria viridis* and *Setaria italica*, model genetic systems for the panicoid grasses. Journal of Experimental Botany.

[ref-50] Li C, Yue J, Wu X, Xu C, Yu J (2014). An ABA-responsive DRE-binding protein gene from *Setaria italica*, *SiARDP*, the target gene of SiAREB, plays a critical role under drought stress. PLOS ONE.

[ref-51] Livak KJ, Schmittgen TD (2001). Analysis of relative gene expression data using real-time quantitative PCR and the 2^−ΔΔ*CT*^ method. Methods.

[ref-52] Lowry DB, Hernandez K, Taylor SH, Meyer E, Logan TL, Barry KW, Chapman JA, Rokhsar DS, Schmutz J, Juenger TE (2015). The genetics of divergence and reproductive isolation between ecotypes of *Panicum hallii*. New Phytologist.

[ref-53] Lv DW, Subburaj S, Cao M, Yan X, Li X, Appels R, Sun DF, Ma W, Yan YM (2014). Proteome and phosphoproteome characterization reveals new response and defense mechanisms of *Brachypodium distachyon* leaves under salt stress. Molecular & Cellular Proteomics.

[ref-54] Ma X, Wang P, Zhou S, Sun Y, Liu N, Li X, Hou Y (2015). De novo transcriptome sequencing and comprehensive analysis of the drought-responsive genes in the desert plant *Cynanchum komarovii*. BMC Genomics.

[ref-55] Ma X, Xia H, Liu Y, Wei H, Zheng X, Song C, Chen L, Liu H, Luo L (2016). Transcriptomic and metabolomic studies disclose key metabolism pathways contributing to well-maintained photosynthesis under the drought and the consequent drought-tolerance in rice. Frontiers in Plant Science.

[ref-56] Mabbitt PD, Wilbanks SM, Eaton-Rye JJ (2014). Structure and function of the hydrophilic photosystem ii assembly proteins: Psb27, Psb28 and Ycf48. Plant Physiology and Biochemistry.

[ref-57] Miao Z, Han Z, Zhang T, Chen S, Ma C (2017). A systems approach to a spatio-temporal understanding of the drought stress response in maize. Scientific Reports.

[ref-58] Miller M, Song Q, Shi X, Juenger TE, Chen ZJ (2015). Natural variation in timing of stress-responsive gene expression predicts heterosis in intraspecific hybrids of *Arabidopsis*. Nature Communications.

[ref-59] Muthamilarasan M, Bonthala VS, Khandelwal R, Jaishankar J, Shweta S, Nawaz K, Prasad M (2015). Global analysis of WRKY transcription factor superfamily in Setaria identifies potential candidates involved in abiotic stress signaling. Frontiers in Plant Science.

[ref-60] Muthamilarasan M, Prasad M (2015). Advances in *Setaria* genomics for genetic improvement of cereals and bioenergy grasses. Theoretical and Applied Genetics.

[ref-61] Pan L, Meng C, Wang J, Ma X, Fan X, Yang Z, Zhou M, Zhang X (2018). Integrated omics data of two annual ryegrass (*Lolium multiflorum* L.) genotypes reveals core metabolic processes under drought stress. BMC Plant Biology.

[ref-62] Pollard M, Beisson F, Li Y, Ohlrogge JB (2008). Building lipid barriers: biosynthesis of cutin and suberin. Trends in Plant Science.

[ref-63] Puranik S, Bahadur RP, Srivastava PS, Prasad M (2011). Molecular cloning and characterization of a membrane associated NAC family gene, *SiNAC* from foxtail millet [*Setaria italica* (L.)P. Beauv]. Molecular Biotechnology.

[ref-64] Puranik S, Sahu PP, Mandal SN, VS B, Parida SK, Prasad M (2013). Comprehensive genome-wide survey, genomic constitution and expression profiling of the NAC transcription factor family in foxtail millet (*Setaria italica* L). PLOS ONE.

[ref-65] Qi X, Xie S, Liu Y, Yi F, Yu J (2013). Genome-wide annotation of genes and noncoding RNAs of foxtail millet in response to simulated drought stress by deep sequencing. Plant Molecular Biology.

[ref-66] Ramachandra Reddy A, Chaitanya KV, Vivekanandan M (2004). Drought-induced responses of photosynthesis and antioxidant metabolism in higher plants. Journal of Plant Physiology.

[ref-67] Redillas MC, Jeong JS, Kim YS, Jung H, Bang SW, Choi YD, Ha SH, Reuzeau C, Kim JK (2012). The overexpression of *OsNAC9* alters the root architecture of rice plants enhancing drought resistance and grain yield under field conditions. Plant Biotechnology Journal.

[ref-68] Reynolds M, Tuberosa R (2008). Translational research impacting on crop productivity in drought-prone environments. Current Opinion in Plant Biology.

[ref-69] Rizhsky L, Liang H, Shuman J, Shulaev V, Davletova S, Mittler R (2004). When defense pathways collide. The response of *Arabidopsis* to a combination of drought and heat stress. Plant Physiology.

[ref-70] Safi H, Saibi W, Alaoui MM, Hmyene A, Masmoudi K, Hanin M, Brini F (2015). A wheat lipid transfer protein (TdLTP4) promotes tolerance to abiotic and biotic stress in *Arabidopsis thaliana*. Plant Physiology and Biochemistry.

[ref-71] Saito R, Smoot ME, Ono K, Ruscheinski J, Wang PL, Lotia S, Pico AR, Bader GD, Ideker T (2012). A travel guide to Cytoscape plugins. Nature Methods.

[ref-72] Shivaprasad PV, Dunn RM, Santos BA, Bassett A, Baulcombe DC (2012). Extraordinary transgressive phenotypes of hybrid tomato are influenced by epigenetics and small silencing RNAs. EMBO Journal.

[ref-73] Singh AK, Kumar R, Tripathi AK, Gupta BK, Pareek A, Singla-Pareek SL (2015). Genome-wide investigation and expression analysis of sodium/calcium exchanger gene family in rice and *Arabidopsis*. Rice.

[ref-74] Singh BK, Sharma SR, Singh B (2010). Heterosis for superoxide dismutase, peroxidase and catalase enzymes in the head of single cross-hybrids of cabbage (*Brassica oleracea* var. *capitata*). Journal of Genetics.

[ref-75] Singh D, Singh CK, Taunk J, Tomar RSS, Chaturvedi AK, Gaikwad K, Pal M (2017). Transcriptome analysis of lentil (*Lens culinaris* Medikus) in response to seedling drought stress. BMC Genomics.

[ref-76] Suzuki T, Masuda T, Singh DP, Tan FC, Tsuchiya T, Shimada H, Ohta H, Smith AG, Takamiya K (2002). Two types of ferrochelatase in photosynthetic and nonphotosynthetic tissues of cucumber: their difference in phylogeny, gene expression, and localization. Journal of Biological Chemistry.

[ref-77] Tang S, Li L, Wang Y, Chen Q, Zhang W, Jia G, Zhi H, Zhao B, Diao X (2017). Genotype-specific physiological and transcriptomic responses to drought stress in *Setaria italica* (an emerging model for Panicoideae grasses). Scientific Reports.

[ref-78] Trapnell C, Williams BA, Pertea G, Mortazavi A, Kwan G, Van Baren MJ, Salzberg SL, Wold BJ, Pachter L (2010). Transcript assembly and quantification by RNA-Seq reveals unannotated transcripts and isoform switching during cell differentiation. Nature Biotechnology.

[ref-79] Vendruscolo EC, Schuster I, Pileggi M, Scapim CA, Molinari HB, Marur CJ, Vieira LG (2007). Stress-induced synthesis of proline confers tolerance to water deficit in transgenic wheat. Journal of Plant Physiology.

[ref-80] Vermeirssen V, De Clercq I, Van Parys T, Van Breusegem F, Van de Peer Y (2014). *Arabidopsis* ensemble reverse-engineered gene regulatory network discloses interconnected transcription factors in oxidative stress. The Plant Cell.

[ref-81] Wang M, Li P, Li C, Pan Y, Jiang X, Zhu D, Zhao Q, Yu J (2014). *SiLEA14*, a novel atypical LEA protein, confers abiotic stress resistance in foxtail millet. BMC Plant Biology.

[ref-82] Wang Y, Li L, Tang S, Liu J, Zhang H, Zhi H, Jia G, Diao X (2016b). Combined small RNA and degradome sequencing to identify miRNAs and their targets in response to drought in foxtail millet. BMC Genetics.

[ref-83] Wang W, Xin H, Wang M, Ma Q, Wang L, Kaleri NA, Wang Y, Li X (2016a). Transcriptomic analysis reveals the molecular mechanisms of drought-stress-induced decreases in *Camellia sinensis* leaf quality. Frontiers in Plant Science.

[ref-84] Warnes GR (2016). gplots: various R programming tools for plotting data. https://cran.r-project.org/web/packages/gplots/.

[ref-85] Werner T, Schmülling T (2009). Cytokinin action in plant development. Current Opinion in Plant Biology.

[ref-86] Yabe T, Morimoto K, Kikuchi S, Nishio K, Terashima I, Nakai M (2004). The Arabidopsis chloroplastic NifU-like protein CnfU, which can act as an iron-sulfur cluster scaffold protein, is required for biogenesis of ferredoxin and photosystem I. The Plant Cell.

[ref-87] Yadav A, Khan Y, Prasad M (2015). Dehydration-responsive miRNAs in foxtail millet: genome-wide identification, characterization and expression profiling. Planta.

[ref-88] Yadav V, Molina I, Ranathunge K, Castillo IQ, Rothstein SJ, Reeda JW (2014). ABCG transporters are required for suberin and pollen wall extracellular barriers in *Arabidopsis*. The Plant Cell.

[ref-89] Yamaguchi-Shinozaki K, Shinozaki K (2006). Transcriptional regulatory networks in cellular responses and tolerance to dehydration and cold stresses. Annual Review of Plant Biology.

[ref-90] Yang C, Ma B, He SJ, Xiong Q, Duan KX, Yin CC, Chen H, Lu X, Chen SY, Zhang JS (2015). *MAOHUZI6/ETHYLENE INSENSITIVE3-LIKE1* and *ETHYLENE INSENSITIVE3-LIKE2* regulate ethylene response of roots and coleoptiles and negatively affect salt tolerance in rice. Plant Physiology.

[ref-91] Yi F, Chen J, Yu J (2015). Global analysis of uncapped mRNA changes under drought stress and microrna-dependent endonucleolytic cleavages in foxtail millet. BMC Plant Biology.

[ref-92] Yong HY, Zou Z, Kok EP, Kwan BH, Chow K, Nasu S, Nanzyo M, Kitashiba H, Nishio T (2014). Comparative transcriptome analysis of leaves and roots in response to sudden increase in salinity in *Brassica napus* by RNA-seq. Biomed Research International.

[ref-93] Yu C, Zhan Y, Feng X, Huang ZA, Sun C (2017). Identification and expression profiling of the auxin response factors in *Capsicum annuum* L. under abiotic stress and hormone treatments. International Journal of Molecular Sciences.

[ref-94] Zhang S, Chen C, Li L, Meng L, Singh J, Jiang N, Deng XW, He ZH, Lemaux PG (2005). Evolutionary expansion, gene structure, and expression of the rice wall-associated kinase gene family. Plant Physiology.

[ref-95] Zhang K, Gan SS (2012). An abscisic acid-AtNAP transcription factor-*SAG113* protein phosphatase 2C regulatory chain for controlling dehydration in senescing Arabidopsis leaves. Plant Physiology.

[ref-96] Zhang ZF, Li YY, Xiao BZ (2016). Comparative transcriptome analysis highlights the crucial roles of photosynthetic system in drought stress adaptation in upland rice. Scientific Reports.

[ref-97] Zhang J, Liu T, Fu J, Zhu Y, Jia J, Zheng J, Zhao Y, Zhang Y, Wang G (2007). Construction and application of EST library from *Setaria italica*, in response to dehydration stress. Genomics.

[ref-98] Zhang LM, Liu XG, Qu XN, Yu Y, Han SP, Dou Y, Xu YY, Jing HC, Hao DY (2013). Early transcriptomic adaptation to Na_2_CO_3_ stress altered the expression of a quarter of the total genes in the maize genome and exhibited shared and distinctive profiles with NaCl and high pH stresses. Journal of Integrative Plant Biology.

[ref-99] Zhang G, Liu X, Quan Z, Cheng S, Xu X, Pan S, Xie M, Zeng P, Yue Z, Wang W, Tao Y, Bian C, Han C, Xia Q, Peng X, Cao R, Yang X, Zhan D, Hu J, Zhang Y, Li H, Li H, Li N, Wang J, Wang C, Wang R, Guo T, Cai Y, Liu C, Xiang H, Shi Q, Huang P, Chen Q, Li Y, Wang J, Zhao Z, Wang J (2012). Genome sequence of foxtail millet (*Setaria italica*) provides insights into grass evolution and biofuel potential. Nature Biotechnology.

[ref-100] Zhang H, Shen J, Wei Y, Chen H (2017). Transcriptome profiling of litchi leaves in response to low temperature reveals candidate regulatory genes and key metabolic events during floral induction. BMC Genomics.

[ref-101] Zhao M, Running SW (2011). Response to comments on “drought-induced reduction in global terrestrial net primary production from 2000 through 2009”. Science.

[ref-102] Zhou Y, Yang P, Cui F, Zhang F, Luo X, Xie J (2016). Transcriptome analysis of salt stress responsiveness in the seedlings of Dongxiang wild rice (*Oryza rufipogon* Griff.). PLOS ONE.

[ref-103] Zwack PJ, Rashotte AM (2015). Interactions between cytokinin signalling and abiotic stress responses. Journal of Experimental Botany.

