# Supplementary figures and images for: Transcriptomic studies reveal a key metabolic pathway contributing to a well-maintained photosynthetic system under drought stress in foxtail millet (Setaria italica L.)
Source: PeerJ. 2018 May 8;6:e4752. doi: 10.7717/peerj.4752 (PMC5947103; doi:10.7717/peerj.4752)

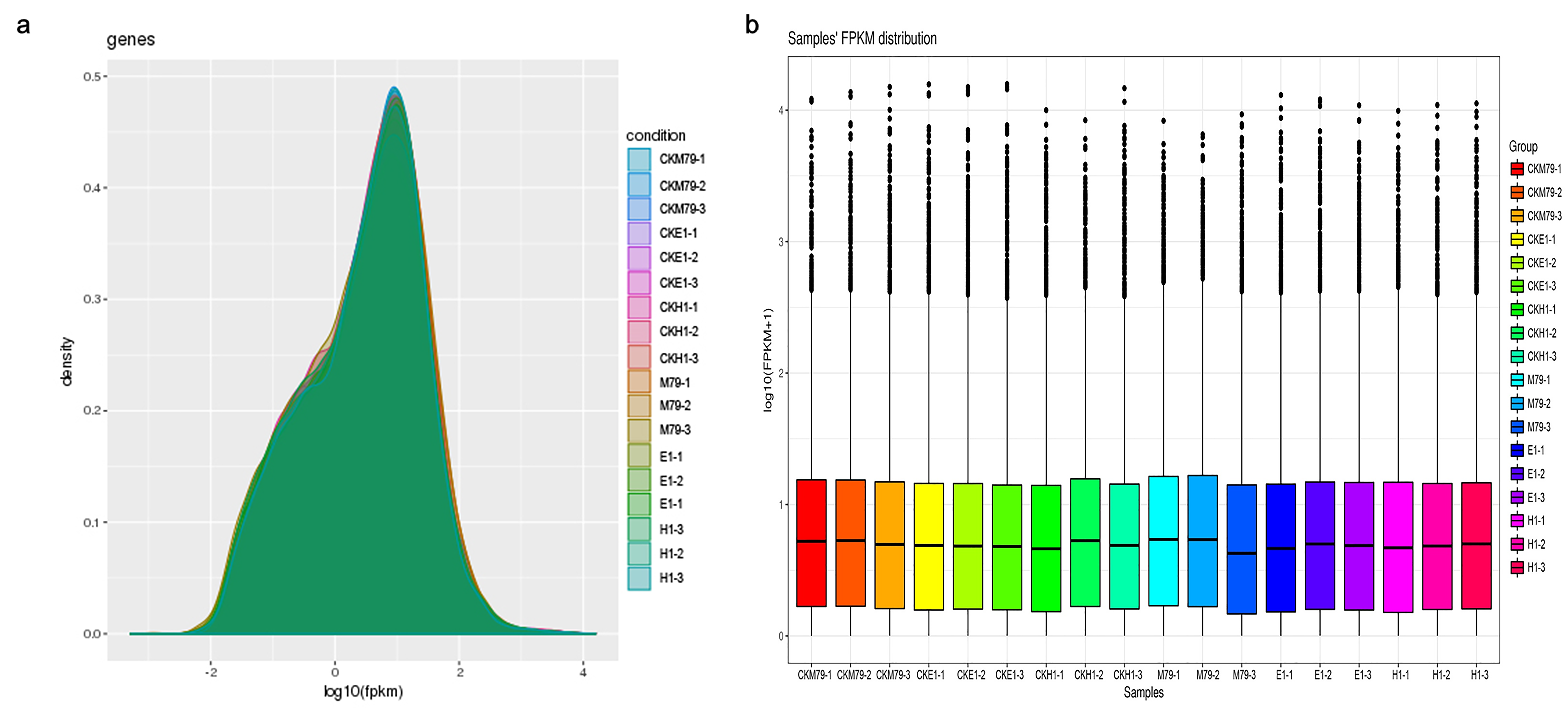

Supplement: Figure S1 — (A) Density distribution; (B) box-plot. [file peerj-06-4752-s018.png]

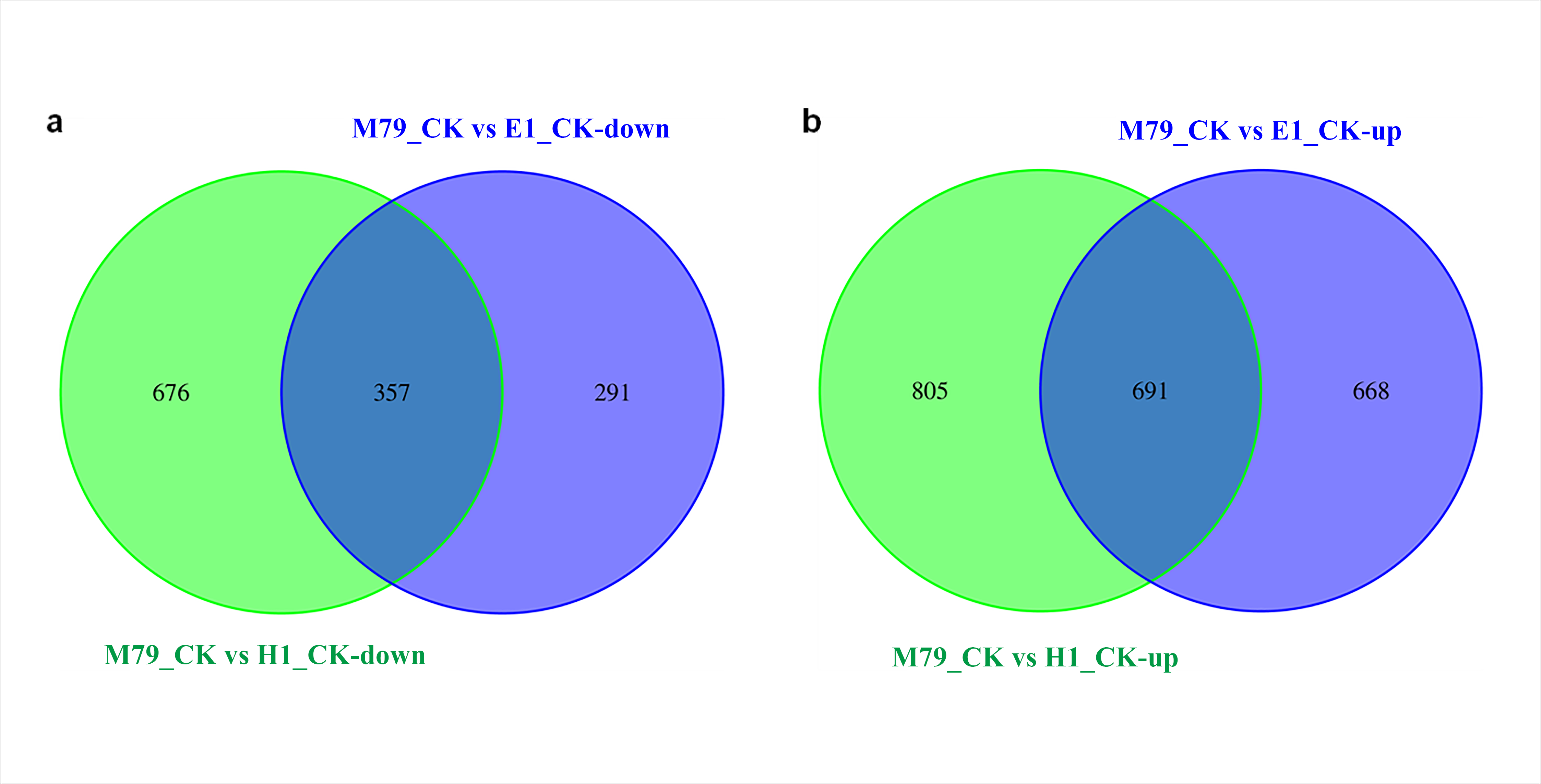

Supplement: Figure S2 [file peerj-06-4752-s019.png]

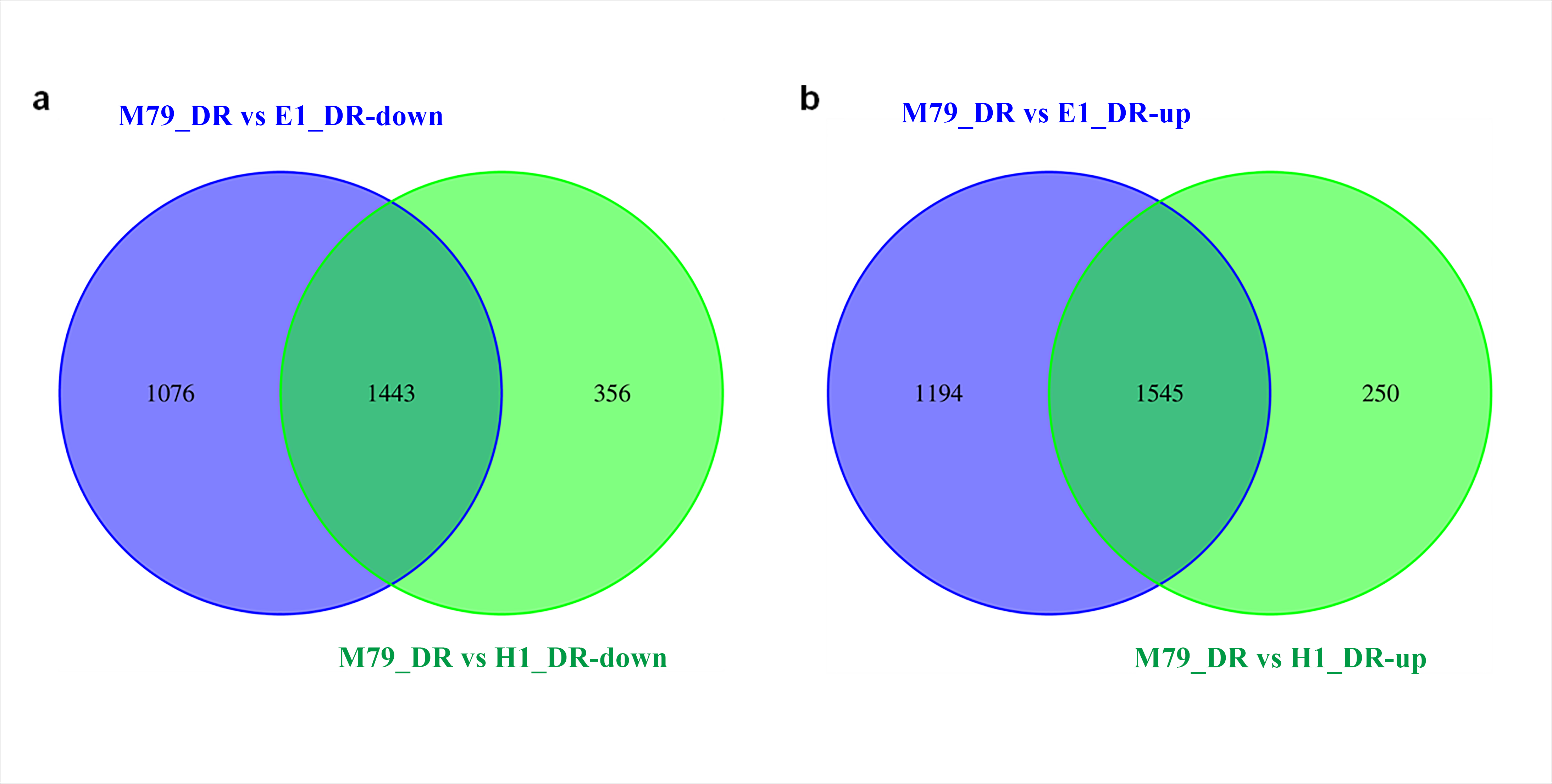

Supplement: Figure S3 [file peerj-06-4752-s020.png]
